# Factors Influencing Usability of a Smartphone App to Reduce Excessive Alcohol Consumption: Think Aloud and Interview Studies

**DOI:** 10.3389/fpubh.2017.00039

**Published:** 2017-04-03

**Authors:** David Crane, Claire Garnett, Jamie Brown, Robert West, Susan Michie

**Affiliations:** ^1^Department of Clinical, Educational and Health Psychology, University College London, London, UK; ^2^Cancer Research UK Health Behaviour Research Centre, University College London, London, UK

**Keywords:** app, digital health, mHealth, alcohol, think aloud, interview study, qualitative

## Abstract

**Background:**

Interventions delivered by smartphone apps have the potential to help drinkers reduce their consumption of alcohol. To optimize engagement and reduce the high rates of attrition associated with the use of digital interventions, it is necessary to ensure that an app’s design and functionality is appropriate for its intended purposes and target population.

**Aims:**

To understand the usability of an app to help people reduce their alcohol consumption.

**Method:**

The app, Drink Less, contains a core module focusing on goal setting, supplemented by five additional modules: self-monitoring and feedback, identity change, cognitive bias re-training, action planning, and social comparison. Two studies were conducted, a “think aloud” study performed with people using the app for the first time and a semistructured interview study performed after users had had access to the app for at least 2 weeks. A thematic analysis of the “think aloud” and interview transcripts was conducted by one coder and verified by a second.

**Results:**

Twenty-four participants, half of whom were women and half from disadvantaged groups, took part in the two studies. Three main themes identified in the data were “Feeling lost and unsure of what to do next,” “Make the app easy to use,” and “Make the app beneficial and rewarding to use.” These themes reflected participants’ need for (i) guidance, particularly when first using the app or when entering data; (ii) the data entry process to be simple and the navigation intuitive; (iii) neither the amount of text nor range of options to be overwhelming; (iv) the app to reward them for effort and progress; and (v) it to be clear how the app could help alcohol reduction goals be reached.

**Conclusion:**

First-time and experienced users want an alcohol reduction app to be easy, rewarding, and beneficial to use. An easy-to-use app would reduce user burden, offer ongoing help, and be esthetically pleasing. A rewarding and beneficial app would provide positive reinforcement, give feedback about progress, and demonstrate credibility. Users need help when first using the app, and they need a compelling reason to continue using it.

## Introduction

Excessive alcohol consumption is a major public health issue (1, 2). Alcohol is responsible for approximately 3.3 million deaths worldwide each year and is a causal factor in over 200 diseases and conditions (1, 3, 4). Face-to-face interventions to reduce alcohol consumption are effective and cost-effective but not widely offered (5–8). Digital behavior change interventions (DBCIs) such as web sites and smartphone apps may be able to overcome some of the barriers associated with the uptake of face-to-face interventions (9–12). Greater use of a DBCI has been associated with more favorable outcomes (13, 14), but DBCIs commonly experience low rates of engagement and apps tend to be infrequently used (15–18). To increase engagement, it is necessary to examine the usability of the DBCI with the target population to ensure that its design and functionality meets user needs (19, 20).

Traditional user testing has tended to focus on the utilitarian or hedonic qualities of a technology (21–25), such as how fun or absorbing a technology is to use (25–30). However, this approach is not entirely appropriate for DBCIs, where the goal is not necessarily to create a technology that is fun or absorbing but rather, one that encourages sufficient engagement with the intervention for the intended outcomes to be achieved (31). A potentially more suitable method is the person-based approach to intervention development (32). The person-based approach melds traditional user testing with a method that seeks to understand not just the hedonic or utilitarian qualities of a technology but also the appropriateness of the component behavior change techniques (BCTs) and the challenges faced or anticipated in adhering to them. In this way, acceptable and feasible BCTs can be identified and improved, with impractical or intrusive BCTs replaced (32).

Usability studies of DBCIs commonly use the “think aloud” method to capture experiences of using technology (33, 34). The method encourages users to verbalize in running commentary what they are looking at, thinking about, doing, and feeling as they engage with the technology spontaneously or in response to researcher-directed tasks (35). “Think aloud” studies can be performed with small numbers of participants (36, 37) who provide information about difficulties encountered using the technology, whether the BCTs appear acceptable or impractical, and what users think of the technology’s graphic design, navigation, and functionality.

“Think aloud” studies are a valuable tool for user testing but are typically not conducted in real-world settings. Smartphones are often used in contexts that present specific challenges to usability, e.g., when walking or on public transport, in noisy or distracting environments, and for brief periods of time (38). Furthermore, while it is useful to conduct studies that evaluate a user’s first impressions of an app, DBCIs often require repeated use in order to influence behavior. The extent to which a user returns spontaneously to the intervention, the degree to which prompts and notifications are intrusive, the suitability of prolonged used of the BCTs suggested, and the ease of interaction in different contexts of use can better be answered after users have engaged with the app for a period of time. Conducting usability studies after users have had the opportunity to use the app repeatedly and in natural settings is recommended (32, 38–40).

The studies reported here assessed the usability of a new app in terms of both immediate impression and experience of use. The first study aimed to assess initial impressions and the ease of using features, entering data and navigating to specific items of content by a “think aloud” study performed with users encountering the app for the first time. The second study aimed to understand the lived experience of the app by a semistructured interview study performed with users who have had access to the app for at least 2 weeks. Both studies adopted a person-based approach in order to determine whether the BCTs used in the intervention are acceptable, easy to use, and feasible and if not, what suggestions for improvement can be gained.

The app to be assessed, Drink Less, is intended to help harmful and hazardous drinkers reduce their consumption of alcohol. Users have access to modules that allow them to set goals, create action plans, monitor their drinking, and engage in a range of tasks designed to help reframe their responses toward alcohol. Feedback is provided on consumption and how this relates to the goals set and to other people in the UK (further information on the modules and their BCTs is in Section “Materials,” below).

Behavior change techniques were selected on the basis of theory and evidence, and many have been used in face-to-face and web-based interventions (41, 42). However, there does not appear to be evidence about whether these BCTs are acceptable to users of an alcohol reduction app, whose small screens and keyboards, and the wide range of settings in which the app is likely to be used, may present particular usability challenges (38, 43–46). There is evidence that users value the BCT of self-monitoring, but are critical of difficulties with entering drinks (47). This finding indicates that simply providing an alcohol reduction BCT is unlikely to ensure engagement; the BCT must also be implemented in ways that people find usable for the specific task at hand. Previous studies of alcohol apps have examined usability in general terms, such as ease of use and helpfulness, but have not examined the implementation of BCTs or detailed what aspects may need to be improved (48–51). Greater understanding of how the BCTs in alcohol reduction apps can be made more acceptable and usable is needed if more effective interventions are to be developed.

Given the huge amount of research on the usability of apps, it is natural to ask, why study usability of an alcohol reduction app in particular? Our rationale for undertaking this study arises from (1) the characteristics and needs of users, and (2) what the app is attempting to achieve.

In terms of the characteristics and needs of users, this is a group motivated to change their behavior and who hope the app will help them do so. This is very different from the case with most apps, which seek to entertain or provide an immediate function. The key reward that users of an alcohol reduction app are likely to gain is a sense of satisfaction at having moved closer toward their goal. There is, therefore, much greater burden on an app to provide intermediate rewards and also to be extremely easy to use in order to increase a user’s persistence.

In terms of what the app is trying to achieve, the assumption is that a certain level of continued engagement with the app is important for success. We do not know what that level is, but it demands a more structured engagement than the kind of “as-and-when” mode of operation of other apps. Typically users have to remember, and be motivated to, initiate a session with the app themselves out of a sense of commitment to the behavior change goal.

Concern has been expressed that DBCIs may exacerbate health inequalities (52), since people with greater social disadvantage tend to have poorer online literacy (53). However, it is an empirical issue, and there are promising results for the effectiveness of DBCIs among disadvantaged groups for other health behaviors [e.g., smoking (54)]. Specific evidence for the effectiveness of apps for alcohol reduction among disadvantaged groups appears to be lacking (42). Few apps seem specifically targeted at disadvantaged groups, and studies that have included people from these groups tend not to report results for them separately (55, 56). Care should be taken to ensure that alcohol reduction interventions are suitable for disadvantaged groups because of the disproportionally negative effect alcohol has on them (57, 58). Including disadvantaged groups in the design and usability testing of new interventions can produce DBCIs that are more appealing to these groups (59). We will therefore recruit half the participants for each study from disadvantaged groups in order the needs of people in these groups are understood.

The aim of this study is to explore user views toward an app to help people reduce their consumption of alcohol and determine whether the BCTs are acceptable and feasible to users and how they might be improved. Findings will not only inform the refinement of the app but, depending on the outcome of the RCT, may also inform intervention developers about how an app’s BCTs and design can be altered to improve usability, reduce attrition, and increase engagement.

## Study 1: Investigation of First Impressions: “Think Aloud”

### Methods

#### Study Sample

Participants were recruited from a convenience sample of members of staff at a London university, their family, and friends, as well as subscribers to an alcohol reduction mailing list. Inclusion criteria were people interested in reducing their alcohol consumption and who had an Alcohol Use Disorders Identification Test-Consumption (AUDIT-C) score greater than 5, which reflects potentially harmful levels of drinking (60). A purposeful sampling approach was taken in order to ensure the views of disadvantaged groups were gathered; half of the participants in both studies had no post-16 educational qualifications, were unemployed, or were employed in a routine/manual occupation. Participants were given £20 in compensation for their time.

Of the 12 participants in the “think aloud” study, 50% were female and 50% were from disadvantaged groups. Their mean age was 42 years, and the mean interview length was 59 min.

### Materials

Five behavior change modules were included in the app: normative feedback, self-monitoring and feedback, action planning, cognitive bias re-training, and identity change. The contents of each module and the registration process are summarized below. Full details of the content of the app can be found in two PhD theses (61, 62).

#### Registration

On opening the app for the first time, users were presented with the 10-item AUDIT, on completion of which the user’s AUDIT score and brief information about what the score indicated were provided. Users were then asked to complete baseline demographic measures, after which registration was complete.

#### Normative Feedback

**Figure 1:**
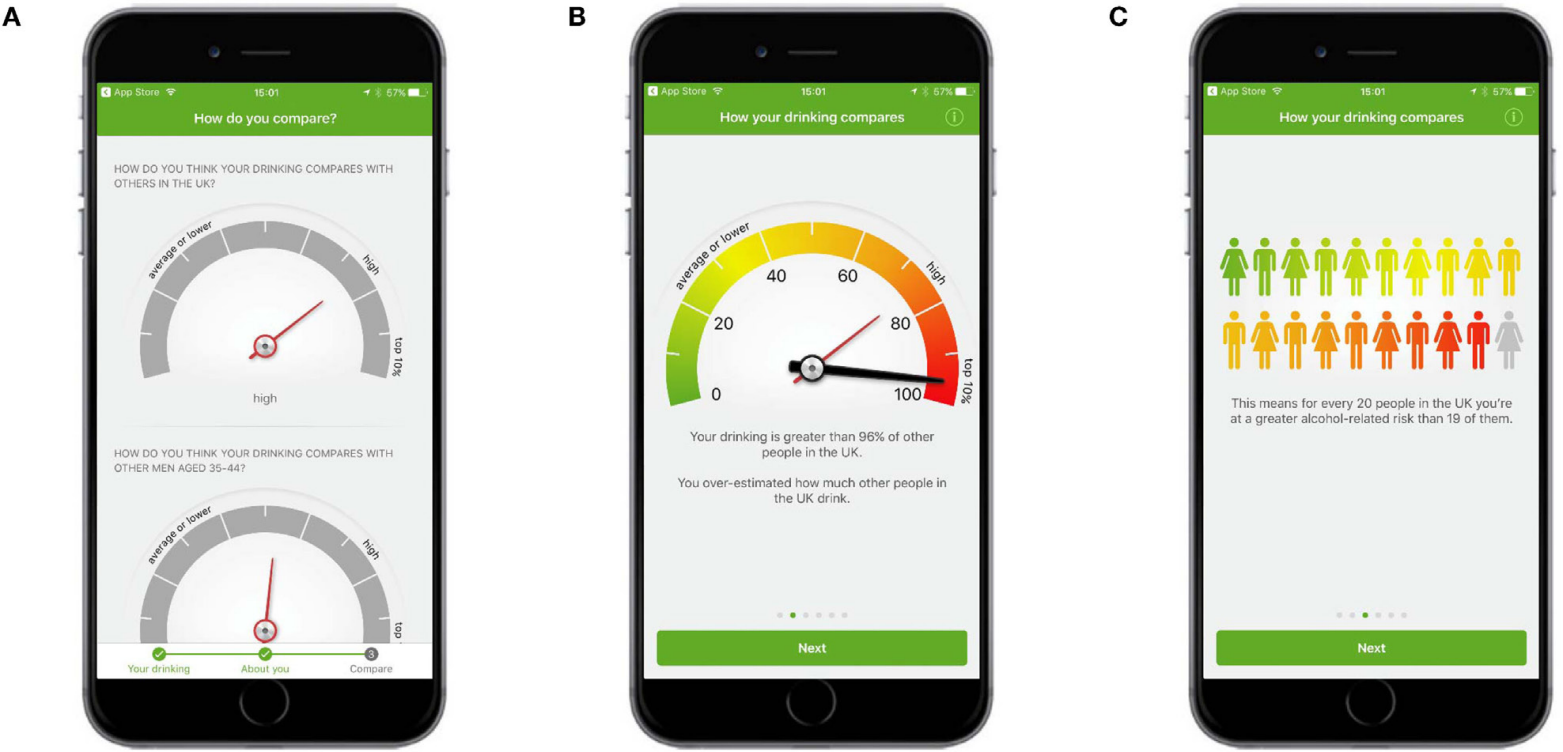
**Normative feedback**. **(A)** Users were asked to indicate how they thought their drinking compared to (1) other people in the UK and (2) other people of their age and gender. **(B)** Feedback showed how a user’s drinking actually compared to people in the UK and people of their own age and gender. **(C)** More feedback showed how a user’s drinking compared to people in the UK and people of their own age and gender.

Following registration, users were asked to indicate how they thought their drinking compared to (1) other people in the UK and (2) other people of their age and gender, using a dial mechanism (Figure 1A). Users were then given feedback which showed how their drinking actually compared to people in the UK and people of their own age and gender, using the same dial mechanism and other graphical representations (Figures 1B,C).

#### Self-Monitoring and Feedback

**Figure 2:**
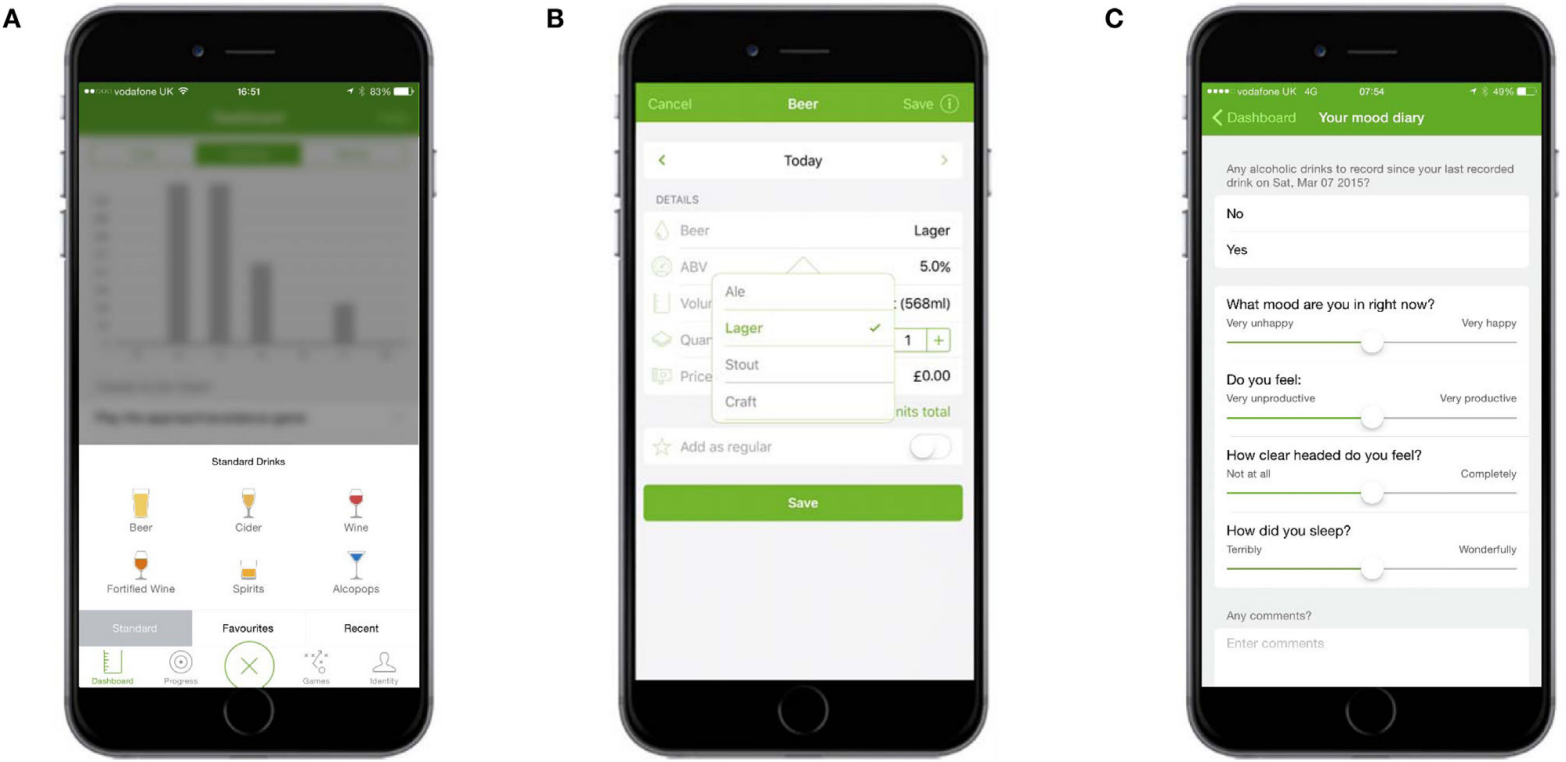
**Self-monitoring and feedback: monitoring consumption and the consequences of consumption**. **(A)** Users could select one of six types of drink … **(B)** … and then chose options for each. **(C)** Users monitored the consequences of consumption by recording daily their mood, productivity, clarity, and sleep quality scores.

**Figure 3:**
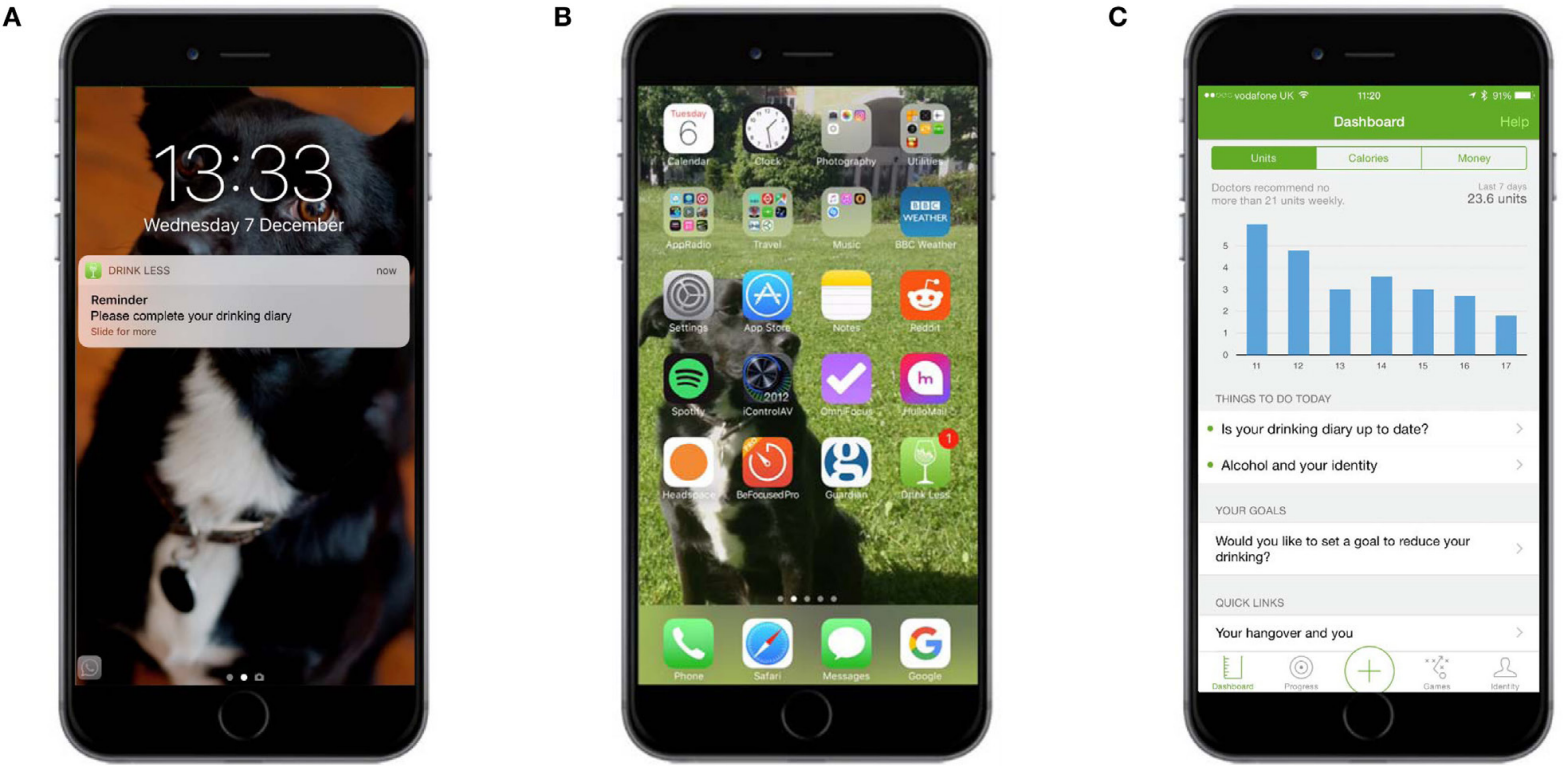
**Self-monitoring and feedback: alerts to monitor consumption and consequences of consumption**. **(A)** Prompt on the user’s home screen. **(B)** Alert on the “badge app icon” (fifth row, last app). **(C)** Alert on the dashboard (in “Things to do today”).

Participants were able to self-monitor their consumption of alcohol and the consequences of consumption. To monitor alcohol consumption, participants tapped a large plus sign in the middle of the navigation bar at the bottom of each screen, choose from one of six drink types, and then choose various options for the selected drink (Figures 2A,B). To self-monitor the consequences of consumption, users recorded a score for mood, productivity, clarity, and sleep quality each morning on a slider (Figure 2C). Users were prompted to record their consumption and their mood scores each day by way of an onscreen alert and message within the app (Figure 3).

**Figure 4:**
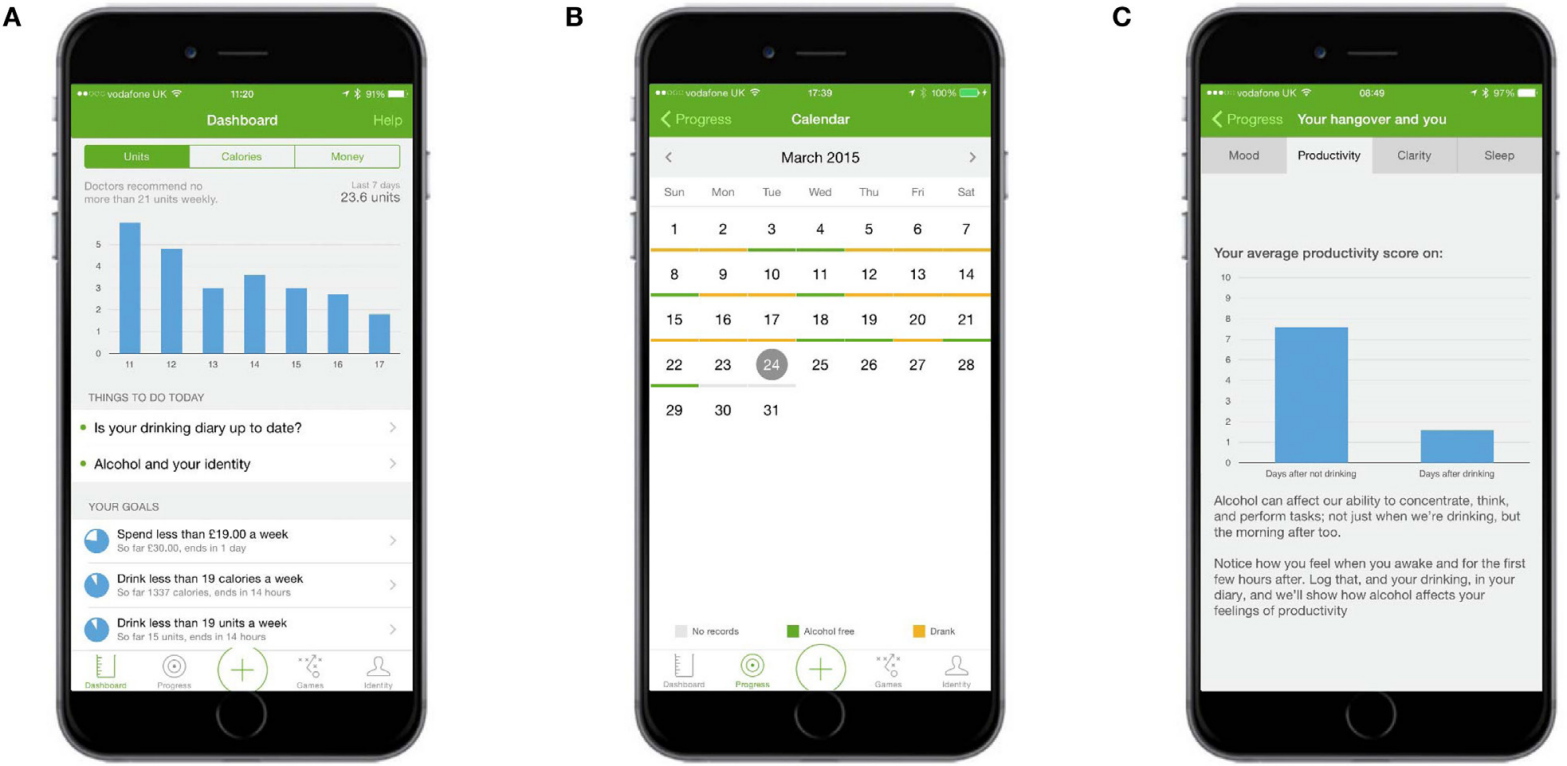
**Self-monitoring and feedback: feedback about consumption and consequences of consumption**. **(A)** Dashboard shows units, calories, and spending graphs as well as summary feedback about progress against goals. **(B)** The calendar provided an overview of a user’s recorded drinks, with days underlined according to whether a user had drunk or not. **(C)** Your Hangover and You presented scores from the mood diary (Figure 2C) in graph form.

**Figure 5:**
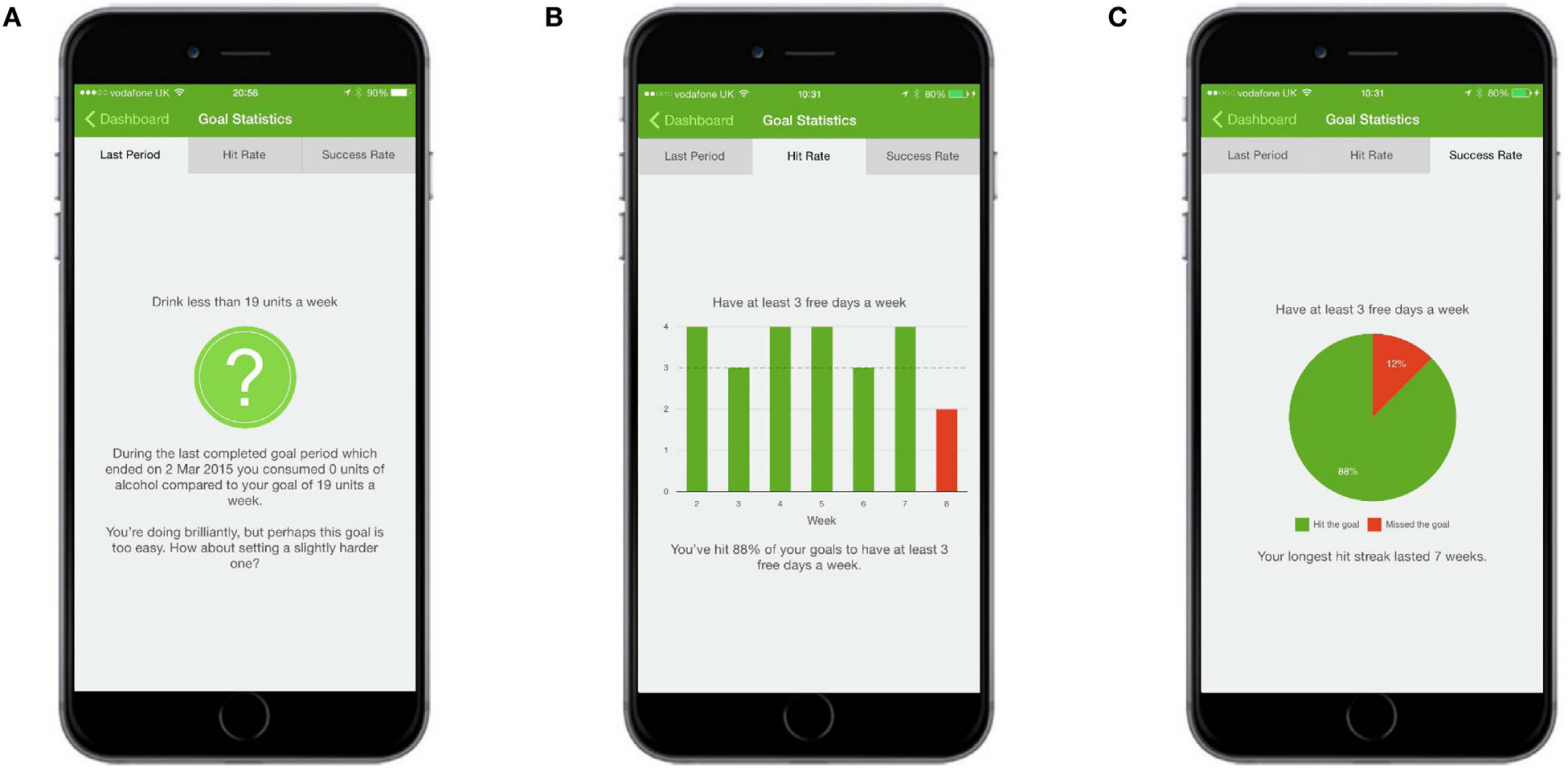
**Self-monitoring and feedback: feedback about consumption**. **(A)** The Last Week screen shows whether a user exceeded, hit, or missed the goal for the previously completed week. **(B)** The “Hit Rate” screen provided an overview of how many times the goal had been exceeded, hit, or missed since the app was downloaded. **(C)** The “Success Rate” screen provided a total of how many times the goal had been exceeded, hit, or missed since the app was downloaded.

Several forms of feedback were provided. The total amount of alcohol (in units), calories consumed from alcohol, and spend on alcohol was displayed in graphs on the dashboard (Figure 4A). The dashboard also displayed summary feedback about progress against goals and provided links to three types of other goal-related feedback: (1) whether the previous week’s goal had been achieved or missed, (2) progress against the goal for each completed week since the app had been downloaded, and (3) a summary of how many times each goal had been achieved or missed (Figure 5). The calendar provided an overview of a user’s recorded drinks (Figure 4B), with each day underlined according to whether a user had drank (colored orange), not drank (colored green), or not made an entry for that day (colored gray). Users could tap any day to see details of drinks entered; these records could be edited, added to, or deleted.

Feedback about the consequences of consumption was presented on the “Your hangover and you” screen, which contained four graphs comparing a participant’s mood, productivity, clarity, and sleep quality on days after drinking with days after not drinking (Figure 4C).

#### Action Planning

**Figure 6:**
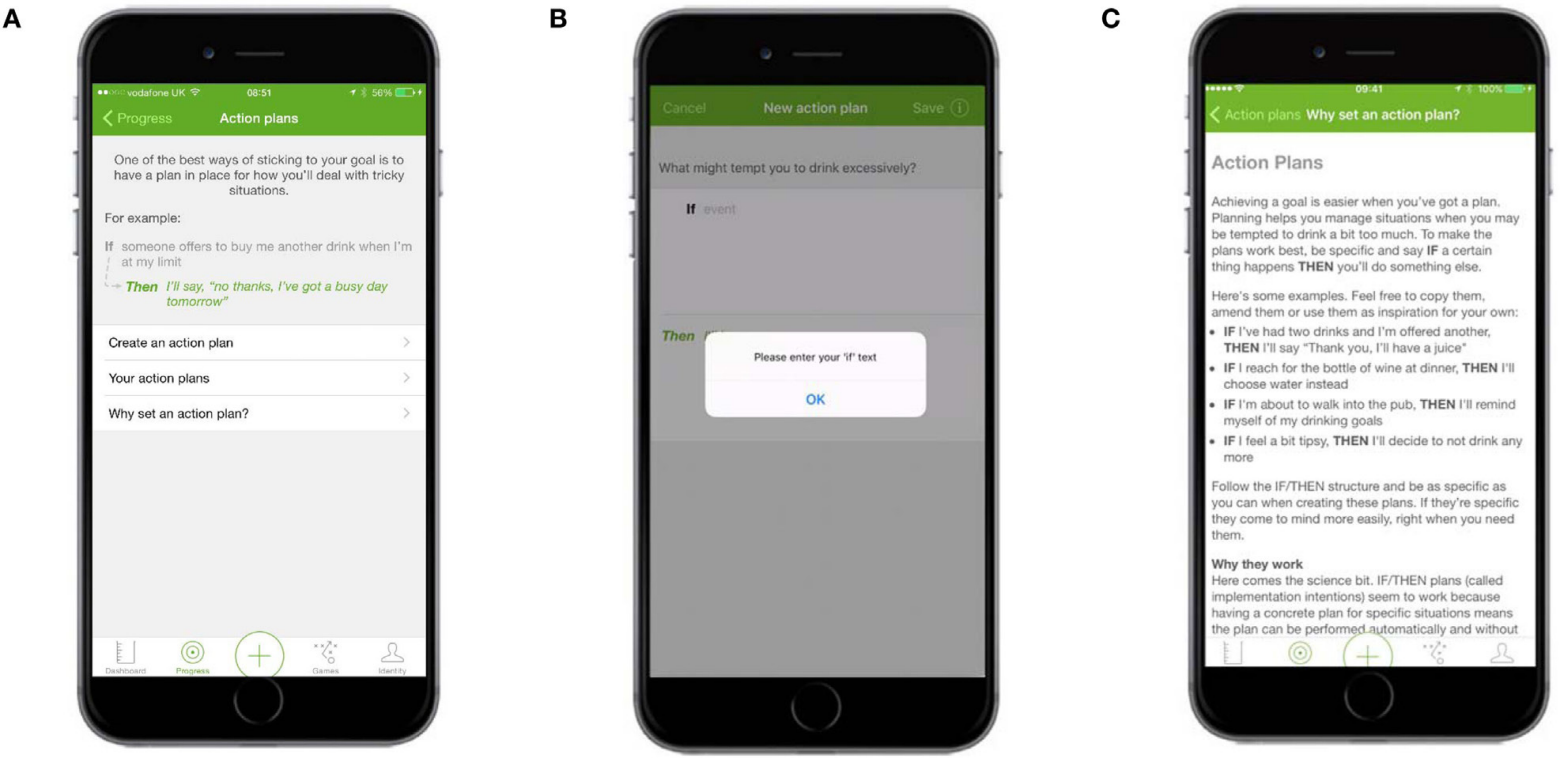
**Action plans—main screen and Why set an action plan**. **(A)** The main Action Plans screen contained information about the benefits of setting an action plan and an example of one. **(B)** The Create an Action Plan screen asked users to fill in two fields corresponding to the If and Then components of an implementation intention. **(C)** Information explaining the benefits of an action plan and examples of action plans.

Action planning was presented within a “Create and View Action Plans” section. At the top of the screen was information about the benefits of setting an action plan and an example of one (Figure 6A). The term “Action plan” was used in place of the more accurate, but potentially less well-understood, “implementation intentions.” The Create an Action Plan screen asked users to fill in two fields corresponding to the If and Then components of an implementation intention (Figure 6B). Other screens displayed the action plans users had already set and provided further information about, and examples of, action plans (Figure 6C).

#### Cognitive Bias Re-Training

**Figure 7:**
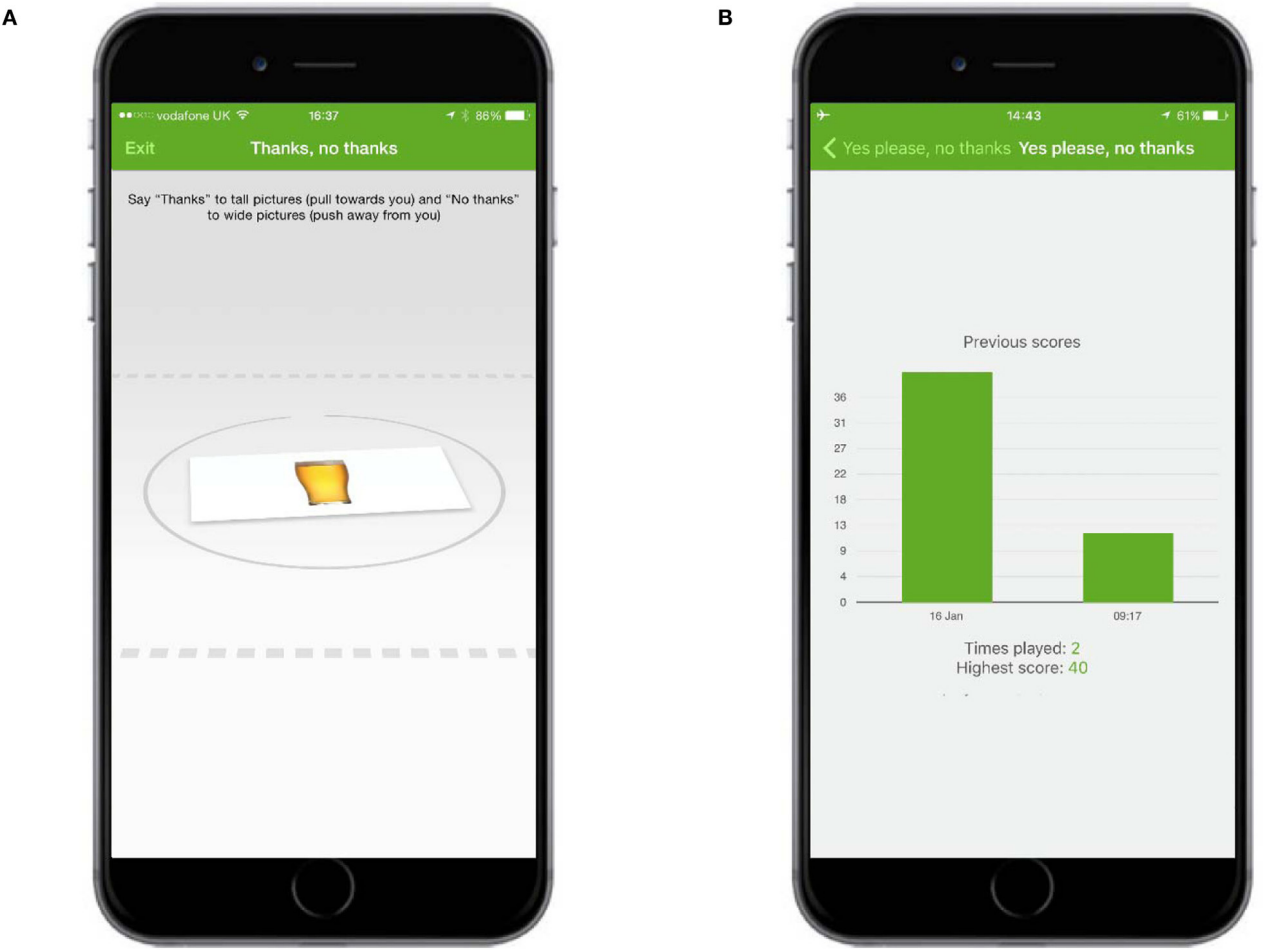
**Cognitive bias re-training**. **(A)** Users were instructed to use their finger to push the alcoholic drink away from them and to pull the non-alcoholic drink toward them. **(B)** Other screens displayed a graph of previous scores over time.

The cognitive bias re-training game presented users with either an image of an alcoholic drink or an image of a non-alcoholic one. Users were instructed to use their finger to push pictures in portrait form (“tall”) away from them and to pull pictures in landscape form (“wide”) toward them (Figure 7A). The total score for each game was the number of images correctly pulled or pushed in a 60-s period. Other screens provided instructions about the game and displayed a graph of previous scores over time (Figure 7B).

#### Identity Change

**Figure 8:**
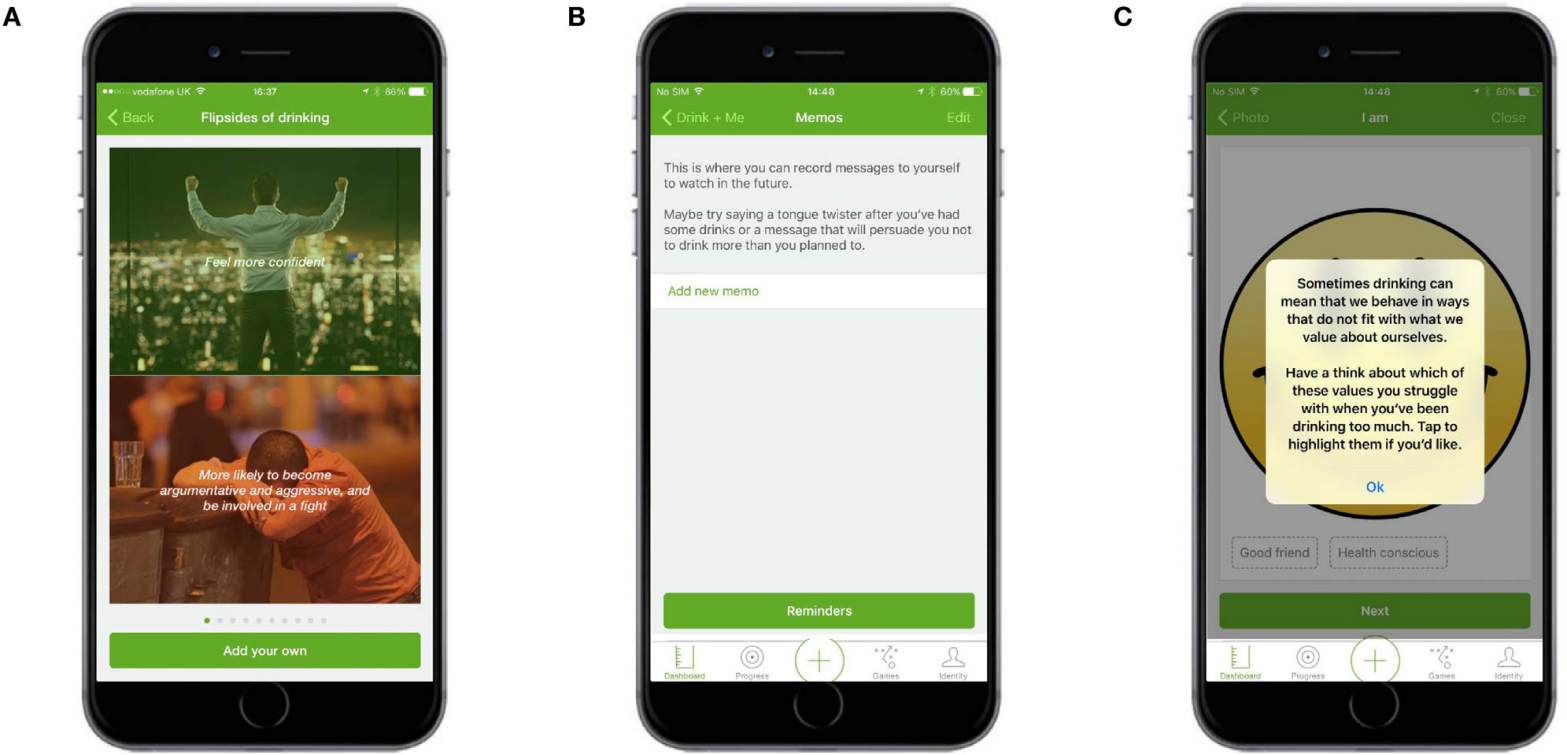
**Identity change**. **(A)** Flipsides of drinking showed images and text representing a positive or benefit of drinking with a negative or cost of drinking. **(B)** Memos allowed users to record video messages to watch at a later date. **(C)** I am … allowed users to select values of importance to them and then reflect on how these values might be affected by alcohol.

The Identity Change section contained three elements: (1) flipsides of drinking, which showed images and text representing a positive or benefit of drinking with a negative or cost of drinking (Figure 8A); (2) memos, which allowed users to record video messages to watch at a later date, for example, they could record a message when sober to remind themselves of their goal during a night of drinking (Figure 8B); and (3) “I am…,” which allowed users to select personal values of importance to them, such as being honest or responsible, and then reflect on how these values might be affected by alcohol consumption (Figure 8C).

#### Goal Setting

**Figure 9:**
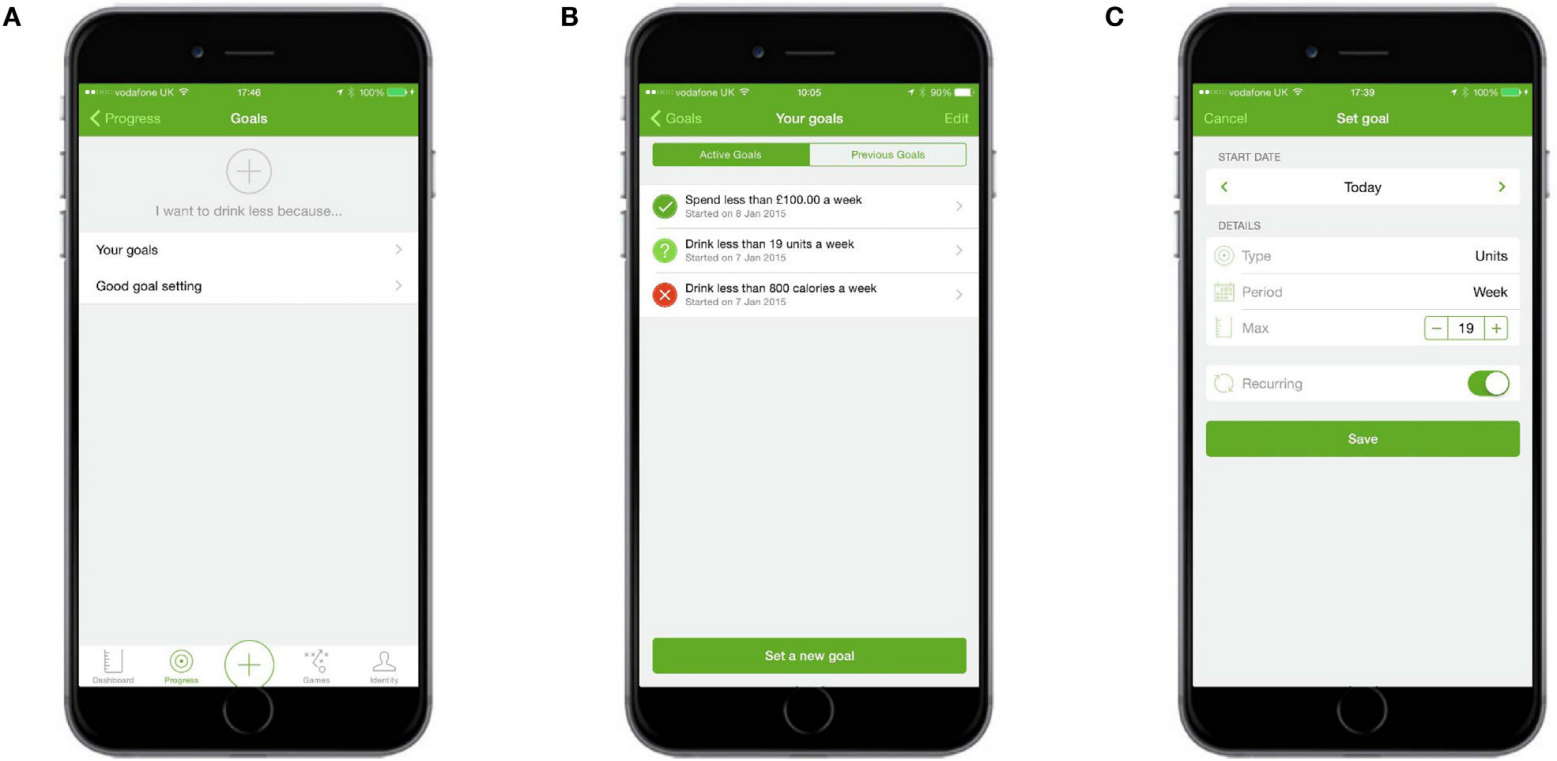
**Goal setting**. **(A)** Users could set an overarching goal for drinking less, create new goals, or get information about good goal setting. **(B)** “Your goals” allowed users to set new goals and see summary feedback about current goals. **(C)** Users could choose unit, spending, calorie, or alcohol-free day goals.

Users were able to set an overarching reason for drinking less and were presented with links to set new goals and view existing ones. They were also given information about how to set good goals. Users could set goals for any combination of the number of units and/or alcohol-free days they wanted to have each week or month, the maximum number of calories, and/or the maximum amount of money they wanted to spend on alcohol each week or month (Figure 9).

### Procedure

Participants were set a series of tasks, for example, complete the registration process, add drinks to the drinking diary, set goals, play the game, and browse the app. They were asked to verbalize what they thinking about, looking at, doing, and feeling throughout the process. After the “think aloud” study had finished, users were asked if they have any suggestions for how the app could be improved or any additional comments they wished to make. A full list of tasks set and questions asked can be found in Appendix S1 in Supplementary Material.

Participants chose the date and time of the interview, and were reassured that their responses would be anonymized and stored securely and that they had a right to withdraw any time. Participants gave written informed consent before the study commenced. All interviews were carried out by the first author and were audio recorded.

Ethical approval was obtained from the Clinical, Educational, and Health Psychology Research Department’s Ethics Committee at University College London (UCL), Reference: CEHP/2013/50, 1st May 2015.

### Analysis

Interviews were audio recorded, transcribed verbatim, and analyzed with thematic analysis, a method commonly used in qualitative research for “identifying, analysing and reporting patterns (themes) within data” [p. 79 (63)]. The method allows for the similarities, differences, and key features of a large body of data to be summarized and for its predominant themes to be identified. Thematic analysis is suitable for mixed-methods qualitative studies (64) and has been used to analyze usability studies of internet interventions and smartphone apps (65–67).

Transcripts were read multiple times in order for their content to be familiarized. Notes taken during these readings were used to generate an initial set of themes. Extracts were coded against these initial themes in an iterative process that led to new themes being identified or existing themes renamed in ways that more accurately captured the essence of the data. Transcripts were reread once coded had finished in order to ensure that all extracts relevant to the research question of understanding user views toward an alcohol reduction app had been identified and that extracts had been coded against the most appropriate theme. Themes were grouped into themes and subthemes and hierarchically organized to reflect their prevalence in the data. Quotes that accurately illustrated the themes were identified. Quotes were edited to improve readability without changing the essence of the quote (unedited transcripts are available from the first author on request). To verify coding accuracy, a second coder independently coded 10% of the extracts, chosen at random, against the finalized set of themes. Percentage agreements were 84% agreement for the first study and 90% agreement for the second.

### Results

Three themes and 12 subthemes were identified, as summarized below.

#### “Feeling Lost and Unsure of What to Do Next”

Participants using the app for the first time frequently expressed confusion about how to use the app and how to navigate through it. Confusion was most pronounced when participants first started using the app after completing the registration process.

#### “Help Me when First Using the App”

Registration is an expected, familiar, and uncomplicated process that participants worked through sequentially. When complete, participants were automatically taken to their dashboard, a screen that contained an empty graph and a number of links to other modules in the app. The abrupt appearance of this screen, its lack of visual concordance with the screens that preceded it, and the number of links available confused the participants, who were unsure if registration had finished and which link they should start with. This created a poor first impression, with almost all participants expressing a desire for a stepped guide to walk them through their initial use of the app.

I want something to tell me “Do number 1 first, then number 2. When you’ve done this go here” so I don’t have to think too much about it. Once I’ve got it up and running I’m fine.

[P1, Female]

I got confused when I’d finished logging-in. There was nowhere to say “Welcome, you’ve registered.” There was nothing that told me I’d finished registering. Which was annoying.

[P12, Male]

#### “How Do I Get to Where I Need to Be?”

Participants often felt disorientated within the app and were unsure how to navigate through it. They were not comfortable exploring the app and clicking links at will, often because they thought there were things they should be doing to set the app up but weren’t clear what these things were. When unsure where to go next, participants tried to retrace their steps and became frustrated when there was no easy or obvious way for them to do so. In the absence of guidance, some participants worked logically through the app, moving left-to-right through the horizontal tab bar at the bottom of the app and top to bottom on the screen. If the order of items did not make sense, if links took participants to an unexpected place, or when the navigation was inconsistent (on some screens the horizontal tab bar was hidden) participants felt confused and annoyed.

Okay, I’ve done my goals. But I don’t know what I do next. Do I press Games, do I press Dashboard again?

[P5, Female]

Okay, so now the mist has gone up again, because it’s not telling me where to go next. There’s no Exit button, there’s nothing.

[P4, Male]

### “Make the App Easy to Use”

Participants wanted a visually appealing app that helped them learn how to use it and did not overwhelm them with choice.

#### “Do Not Make Me Work”

Participants wanted an app whose use required minimal effort. Some said they may be willing to invest more time than they would with other apps because this app was designed to help them. Others said they would stop using the app if it was too difficult, despite believing that their drinking was an issue they needed to address. Participants had formed expectations about how elements of the app should work based on their experience of using other apps and were disappointed when the app failed to meet these expectations (for example, users expected a calendar to appear when a date was tapped). Elements that were straightforward and intuitive, such as adding drinks, were praised.

What I’m thinking is, this better be easy, because otherwise I’m probably not going to do it. If there are too many obstacles in the way I won’t. Even though I know I need to do this, I probably won’t.

[P1, Female]

There was frustration but I wouldn’t just bin it because I know it’s an app that is trying to help me. It probably needs a little bit more time, and I’d be willing to do that.

[P7, Male]

#### “Provide Clear Guidance Throughout”

Guidance was sought when using many other areas of the app, particularly when using modules that required input but came without instruction, for example, setting goals, adding drinks, creating action plans, or using the identity section. Participants often hesitated before entering information, partly because they were unsure what was required of them, partly because they felt the accuracy of their entries was important and did not know if mistakes could be corrected, and partly because they wanted more help from the app about what entries were appropriate (for example, some participants wanted to know whether the goals they had set were realistic). Participants were frustrated when the app prevented them from completing tasks, such as saving an action plan, without clear indication about what they were doing wrong. Instructions provided on how to play the game were thought overly complex and difficult to follow.

So I guess that’s the kind of information I was crying out for when I was doing the goals. How do I set good goals? Is *[spending a maximum of]* £40 unrealistic at this stage?

[P7, Male]

What’s annoying is that I’m really happy that I opened up and put my real reasons, but now I can’t save it because you can’t save unless you put an action in. But if I knew how to take the action I wouldn’t be using the app. Now I’m getting frustrated. Tell me! I want it to tell me.

[P1, Female]

#### “Make It Visually Appealing”

The visual appearance of the app played an important role in its perceived ease of use. Visually unattractive screens were off-putting to participants, who often expressed a desire for more graphic ways of presenting information. Participants found icons more pleasing and more memorable than text links and requested they be used more frequently. Some of the graphs did not make unintuitive sense at first and participants suggested better ways to be found of displaying these data. Screens that were clean and simple were praised and held in contrast to those that were busy and esthetically dull. Many participants appreciated the consistent design of the app, but the green color used throughout was not universally liked.

The drink panel was easy to use because it was really visual.

[P8, Female]

I’d probably like to see a page with icons on rather than text. Because it always feels a bit more serious when you’ve got the text.

[P11, Male]

#### “Do Not Overwhelm Me”

The range of modules available was overwhelming for some participants who wanted a leaner and more condensed app. Screens full of text, or text that appeared complex to read and understand, were off-putting to participants who wanted to keep their reading to a minimum.

First of all, this is a wall of text so it’s not that inviting

[P3, Female]

There seems to be too much on there, I think I would find it off-putting. If I was going to use something it needs to be quick and straightforward. There seems to be too much, too many pages of things to do, which I know that I probably wouldn’t end up doing.

[P10, Female]

#### “Blame Myself, Not the App, If It’s Too Hard to Use”

When a minority of participants did not understand what was asked of them, or did not know how to use the technology, their tendency was to blame themselves, and their shortcomings rather than the app for its poor design.

I’m sure my six year-old nephew would be able to do this by now

[P2, Female]

I’m always my own worst critic. Realising I can’t do this makes me think that I’m at fault, not the app.

[P9, Male]

### “Make the App Beneficial and Rewarding to Use”

Participants did not understand how some of the modules could help them reduce their consumption of alcohol and wanted to know why they should trust the information provided. They sought messages of congratulations and encouragement for actions they had taken and thought the app unrewarding to use when its tone was judgmental or formal. Instead, participants wanted the app to use language that was more friendly and funny.

#### “How Will It Help Me?”

Participants thought the app potentially useful overall but did not understand the benefit of using some of the individual modules, especially the cognitive bias re-training game and the identity change sections, where the relationship between use of the module and reducing alcohol consumption was unclear. The effectiveness of the game was particularly doubted; many participants were unsure of its purpose, or thought it simplistic and unlikely to work. Participants were unlikely to use modules they could see no obvious benefit to and expressed a desire for more information about why a module had been included and how it was theorized to work.

You have finished the game. What was the point of that? Seriously. Really, what was the point of that? Am I missing something? No, I’m not impressed, I don’t know what it was, I don’t know why I’ve just done that.

[P12, Male]

Actually I think more explanation about the psychology around why this might help as a training game would be really useful.

[P8, Female]

#### “Reward Me for My Achievements”

Participants were often unsure if they had successfully completed a task and expressed a desire for visual or audible confirmation at the point of task completion, for example, when a goal had been set. Participants often requested more positive reinforcement from the app and were appreciative when it congratulated them for actions. The sound that was played when participants recorded a drink was particularly appreciated as it was felt to be a reward for their achievements and helped establish a positive relationship with the app.

There’s nothing saying “Right, thank you for that. Next option.”

[P4, Male]

The big green continue at the bottom and when it moves on to the next thing I feel great, I’ve achieved something, I’ve filled something in correctly. I like that. And a nice little noise which made me think, Oh, I’m not an idiot.

[P9, Male]

#### “Do Not Be Judgemental”

Some participants felt the app delivered information in a straightforward and non-judgmental tone. Others took the opposite view and considered the information to be judgmental or preaching, a tone they strongly disliked and which made use of the app feel dissatisfying. The feeling of being judged was often expressed when participants received feedback about their levels of drinking which contrasted with their perception of their consumption, for example, when they received their AUDIT score or were given normative feedback (where participants were shown how their drinking compares to other people in the UK). Participants who greatly underestimated their levels of drinking compared to others found the comparison shocking and thought the app was placing them in a category of drinkers to which they felt they did not belong. It was notable that once participants saw one module of the app as judgmental, they tended to see other modules as judgmental too.

It didn’t make me feel judged. Aside from one or two words here and there it was understanding. I think the tone is understanding.

[P8, Female]

#### “Be Friendly and Funny”

Participants disliked when text was perceived as overly formal or impersonal. They wanted the app to have a friendly, humorous, tongue-in-cheek, and light-hearted tone of voice, despite the serious nature of the subject. A too formal tone was perceived as judgmental and off-putting. Participants appreciated parts of the app that were more light-hearted and said it helped them feel relaxed and made them want to engage more with the app.

The language is a bit stale. It could be more personal.

[P8, Female]

I suppose *[informal language]* is a slightly cheeky, jokey, way in. Of maybe making me feel a little bit more relaxed, maybe not feeling too conscious about giving all my drinking secrets away.

[P1, Female]

#### “Tell Me I Can Trust the App”

The credibility of the app and the information delivered by it was an issue for a number of participants, particularly those who felt their normative feedback had judged them harshly and who then expressed a distrust of data about other people’s drinking. Participants found that credibility was established by use of the UCL logo on the first screen they saw after installing the app and by referencing of studies within the app. A number of participants said that the academic nature of the app and the fact that their data would be part of a study increased the trustworthiness of the information and their positive views toward the app.

I don’t believe that one iota. Less than a pint a day is 85% more than people in Great Britain drink. I don’t believe that for a moment. Either other people are lying, which I assume they might with something like this, or it’s skewed to scare me.

[P9, Male]

I think the UCL thing is quite important, that it is actually coming from academics. One of the things I really liked is when you go into the information and it shows you the research, that gives it some gravitas. I think that gives the app a lot more credibility.

[P5, Female]

## Study 2: Investigation of the Experience of App Use: Semistructured Interviews

### Study Sample

Participants were recruited from users who had downloaded the app from the iTunes Store and volunteered their email address when completing the app’s registration process. Inclusion criteria were the same as for Study 1, with the additional requirement that participants need to have downloaded the app at least 2 weeks prior to the interview taking place. A purposeful sampling approach enabled the views of disadvantaged groups to be gathered; half of the participants were required to have no post-16 educational qualifications, or be unemployed, or be employed in a routine/manual occupation. Participants were given £20 in compensation for their time.

Of the 12 participants in the semistructured interview study, 50% were women and 50% were from disadvantaged groups. Their mean age was 40 years.

### Procedure

Participants were asked a series of semistructured interview questions with a mean interview length of 33 min. Topics included the following: how they found the registration process, what their first impressions of the app were, how easy or difficult they found the app to use, and whether they had any suggestions for how it could be improved or any additional comments they wished to make. A question was added in response to feedback from the first study. A number of users in the first study said they thought normative feedback about their drinking, which compared their drinking to other people in the UK, was not credible. In order to determine the extent to which this view was shared by people in the second study, participants were asked specifically to recall what their response was to the normative feedback. A full set of interview topics is in Appendix S1 in Supplementary Material.

As with Study 1, participants chose the date and time of the interview, and were reassured that their responses would be anonymized and stored securely and that they had a right to withdraw any time. Participants gave written informed consent before the study commenced. All interviews were carried out by the first author and were audio recorded.

### Analysis

Data were analyzed using the same procedure as described for Study 1.

### Results

The themes identified were broadly similar to those identified in the first study. However, the theme most prominent in the first study: “Feeling ‘lost’ and unsure what to do next,” was not identified in the second study. The two other themes from the first study: “Make the app beneficial and rewarding to use” and “Make it easy to use,” were also predominant in the second study, albeit with some different subthemes emerging.

#### “Make the App Beneficial and Rewarding to Use”

As with the first study, participants wanted an app that engaged them and provided clear reasons to continue using it. However, this study revealed that for many participants, the engaging elements were either missing or not apparent. Participants felt dissuaded from using the app when it adopted a judgmental tone of voice and wanted to know that the time and emotional investment they were making would be worthwhile.

##### “How Will It Continue to Help Me?”

Participants thought there was little within the app that would encourage repeated use and either never used, or had stopped using, modules they thought offered no benefit. As with the first study, this was particularly true of the cognitive bias re-training game and the identity change modules, where it was unclear how the module could help reduce alcohol consumption. The self-monitoring and feedback module was thought to have most benefit, and a number of participants used the app for this feature alone, although some said they would prefer to use an app such as MyFitnessPal that allowed them to self-monitor their food intake as well.

I think that’s where it let itself down for me. Once I’d played with it, once I tried the game, done the identity and whatnot, there wasn’t much else there for me.

[P4, Female]

So in the end I reverted back to one app. It may not necessarily provide something I want, it was just a lot more convenient. I drink a wide variety of drinks and I don’t necessarily always know the content. And with MyFitnessPal you can just scan the barcode.

[P10, Male]

##### “Reward Me for My Achievements”

Participants appreciated positive visual and audible confirmations of their actions and achievements. They liked the sound played after a drink has been entered, the green tick that appeared when an alcohol-free day has been recorded, and the green lines under the calendar that show periods of abstinence. Many participants asked for more encouragement and positive reinforcement in the form of badges or smiley faces to indicate periods of success, and supportive messages to encourage drinking reduction.

Then when you say ‘drink free day’ the app goes ‘Congratulations!’ and I feel great.

[P6, Female]

I know this sounds really pathetic but if you could earn badges for your non-alcoholic days, that might make people a bit more focussed on actually not drinking because they know they’re going to earn points.

[P4, Female]

##### “Update Me on How I Am Doing”

Participants wanted to receive feedback about their drinking and how it was changing over time. However, they often could not find this feedback, a situation they found frustrating and demotivating. Some participants had stopped entering data into parts of the app because without feedback, entering data was an unrewarding task. There were requests to make the feedback more prominent, and the app was compared negatively with apps where feedback was easier to find. Participants who managed to locate the feedback appreciated it, though they asked for more encouraging and positive messages.

But one thing that’s a bit strange is you can set goals but there’s never any feedback about whether you’ve made it or not.

[P6, Female]

I couldn’t find any graph that’s reflected the mood so therefore I didn’t see the point of having to fill that part out and I stopped filling it out

[P7, Male]

##### “Do Not Be Judgemental”

As with the first study, some participants saw the app as an impartial tool that did not make judgments about their drinking. Others perceived the app’s agenda was to get them to stop drinking, believed some of the AUDIT questions were overly personal, felt guilty if they had not completed the daily tasks set by the app, and saw the language used as sometimes patronizing. Participants also worried about other people judging them and wanted to keep their use of the app private. They worried that the daily prompt to complete their drinking diary might be seen by colleagues or friends and were concerned that people such as their boss might gain access to their drinking data.

“You should drink less” was quite abrasive to me but potentially that’s the objective if you are trying to get people to drink less.

[P10, Male]

I don’t think it’s made me feel guilty, I think it’s made me feel very conscious of what I’m doing.

[P2, Female]

##### “Tell Me I Can Trust the App”

There were mixed views about the credibility of the normative feedback information, which compared a participant’s drinking to other people in the UK. Some participants found the feedback untrustworthy and thought other people must have underreported how much alcohol they consume. Others valued the comparison as it shocked them into action. In general, the normative feedback information was more trusted than in the first study. However, as participants for this study had searched for and downloaded an alcohol reduction app, it is likely they felt their drinking was problematic and may not have been as surprised to learn it was comparatively high to other people in the UK. Some participants liked that the app was linked to an academic study, appreciated the references that were included, and thought more information about the reliability of the information would further support the credibility of the app and its modules.

I didn’t really believe it either. I thought ‘Wow other people must lie’ because it said I drank more than 95% of the female population and I was thinking ‘There’s no way that’s true’.

[P6, Female]

The reason *[for choosing the app]* was that it was linked to an academic study, it had people behind it who were identifiable, it had some kind of purpose which was bigger than just the app itself. That was the probably the strongest attraction I had to it.

[P1, Male]

#### “Make the App Easy to Use”

As with the first study, participants wanted a visually appealing app that made minimal demands on them and provided guidance about how to use the modules.

##### “Do Not Make Me Work”

Participants in the second study tended to report that the app was easy to use. This was particularly true for the self-monitoring and goals modules, both of which were said to be simple and straightforward, in part because they did not require a great deal of typing. Participants encountered few difficulties with the registration process; some even said they appreciated its comprehensiveness as they felt the app needed to ask a lot of questions in order to be able to help. Participants were disappointed when their expectations about how the app would work and expectations formed from using other apps were not realized. Modules that were new to participants, such as the action plan and cognitive bias re-training game, were not intuitive and a bug that caused the mood diary to record drinks for the wrong day was seen as annoying.

*[The app]* was quite simple and sleek and straightforward. The worst apps are things that make it too complicated or take a long time to fill in.

[P3, Female]

When you enter a drink it’s very easy to vary and be precise. For example, say you’ve got beer you’ve got variations on alcohol content, variations in size. It’s very flexible that is, so you can be accurate.

[P1, Male]

##### “Provide Clear Guidance Throughout”

Participants in the second study reported much less need for guidance on how to use the app. However, confusion remained about a number of modules where input was required but instruction was lacking. Participants wanted more examples and clearer guidance in order to resolve their uncertainty about what constituted an effective action plan or realistic goal. Instructions about how to play the game were considered unclear, and the game itself was not self-explanatory. Participants also requested guidance on how to get the most from the app, for example, they wanted the app to recommend the mood diary be completed at the same time each day in order to make the data more accurate. Some of the graphs were seen as unintuitive, and advice on how to delete drinks or enter drinks for different days was requested.

I think it was quite hard to begin with, not in terms of the app usage itself but creating goals. I found that quite tricky. Maybe if there had been some suggestions about what goals I should have been setting that would have been really useful.

[P8, Female]

I think really I need to play with it more. It’s not self-explanatory to me how you actually fill in some of the bits.

[P4, Female]

##### “Make It Visually Appealing”

The visual appeal of the app was positively commented upon by many participants in the second study who thought the app looked friendly, trustworthy, and non-intrusive. The simple, clean, and clear design, use of green as the main color, the calendar, and the app icon were all liked by participants. Some found the app little dull to view and wanted more imagery, but these were fewer in number than in the first study.

I think generally it’s very well designed. It’s clear, it’s useful. I like the design. I quite like the way it’s all mapped out, I think it’s very good.

[P7, Male]

I liked the way it looked. It felt quite friendly. Not intrusive and not scary I suppose. The colours I liked. They weren’t judgemental colours, there wasn’t a lot of red, so it was quite a safe feeling in terms of the colours that were used.

[P8, Female]

## Discussion

Participants using an alcohol reduction app for the first time and participants who had been using the app for at least 2 weeks wanted an app that was both easy and rewarding to use. While these findings are perhaps unsurprising, few people are likely to want an app to be difficult or unrewarding. The contribution this study makes is to increase understanding of the particular ways in which an alcohol reduction app could be made easy and rewarding to use; findings may be applicable to other apps aiming to promote self-directed behavior change.

### Make the App Easy to Use

The finding that participants wanted an alcohol reduction app to be easy to use accords with a considerable literature about the importance to users of simplicity. The Technology Acceptance Model, a theory of the factors that determine use of a technology, posits that people accept or reject a technology based on how easy to use and how useful they perceive that technology to be (68). Users frequently experience difficulty with new technology (69) and consider ease of use an important and desirable criterion for DBCIs (70). Ease of use affects users’ perceptions of, satisfaction with, and intention to use DBCIs (71), moderates continuing engagement with DBCIs (72, 73), and may influence the perceived credibility of health information delivered digitally (74).

Ease of use for our participants meant that the app needed to do more than reduce user burden, as important as that is (75, 76). Participants often hesitated before entering information, not because the process itself was difficult but because they wanted to enter the “right” information and were concerned their entries might not be changeable. They understood that the app’s ability to help was at least partly dependent on the accuracy of their input and were keen to ensure they correctly recorded consumption, set realistic goals, and created effective action plans. For participants, an easy-to-use alcohol reduction app told them what action was required, gave guidance about how fields should be completed, provided recommendations about, or offered examples of, suitable entries, and made clear how these entries could be edited. The effectiveness of DBCIs may be enhanced when users are given guidance and direction about how to enact the behavior (Crane et al., in preparation). Findings from this study suggest that users may also benefit from guidance and direction about how to engage with the technology.

Ease of use was enhanced when the app was esthetically pleasing. Visually unattractive screens or those heavy with text were described as off-putting; screens with more imagery were praised. Ease-of-use criteria were also applied to the type of imagery used; some participants found graphs difficult to interpret and preferred data to be displayed in more a simple form by, for example, showing the calories consumed from alcohol in a figure, with a separate figure showing how that differed with the previous week. An esthetically appealing app can not only increase ease of use but can also enhance the perceived trustworthiness of the information provided. Participants who liked the design of this app said it seemed friendly and safe. A study of how web-based health information was appraised saw a professional design as indicating credibility to users (74). The skills needed to create a visually appealing app fall outside the traditional expertise of behavioral science researchers (77), but the value placed on design by users emphasizes the need for expert involvement in the design of DBCIs.

The importance of making the app easy to use was illustrated by participants who seemed resistant to change. These participants were interested in reducing their consumption of alcohol (it was an inclusion criterion for the first study and participants in the second study had searched for and downloaded an alcohol reduction app of their own accord). However, it appeared they could be easily dissuaded from using an app to help by relatively minor ease of use issues. Resistance to change can be overcome in therapeutic settings through the creation of a “working alliance,” formed when the client perceives the therapist as an ally who can help (78). Findings from this study suggest that ease-of-use issues may create the impression that the app is not an ally, cannot be relied upon, and so can be discarded. Resolving ease of use issues may strengthen the relationship between user and app, which could result in more effective interventions (79).

### Make the App Beneficial and Rewarding

The Technology Acceptance Model defines the perceived usefulness of a technology as “the degree to which a person believes that using a particular system would enhance his or her job performance” [p. 320 (68)], a definition which reflects the workplace origins of the model. Findings from this study suggest that users of an alcohol reduction app want their technology to be more than just useful. Their needs are for an app that is both beneficial and rewarding.

Health behavior change can often seem an unrewarding process with immediate costs and remote benefits. Behavior change is also an often unsuccessful process; most attempts to eat better, exercise more, stop smoking, or drink less alcohol are not maintained long term (80–83). Unsuccessful attempts to maintain behavior can lead to increased negative affect and decreased self-efficacy (84, 85), which can result in disengagement from goal pursuit (86, 87). Theories such as Thorndike’s Law of Effect, Operant Learning, and Rothman’s Model of Behaviour Maintenance propose that to promote prolonged goal pursuit and encourage maintenance of a new behavior, it may be necessary to positively reinforce change and make salient the beneficial outcomes achieved (88–90).

Users want apps that are rewarding to use (91, 92) and delete those they find difficult, unhelpful, annoying, or burdensome (76, 93, 94). Smoking cessation, and healthy eating and physical activity apps often seek to provide users with a gratifying experience, either by making use of the app intrinsically rewarding or through positive reinforcement of effort or progress (95–97). Providing positive feedback as a reward for behavior is considered important for persuasive technologies (98). However, alcohol reduction apps tend not to use reward BCTs (99); findings from this study suggest that may be an omission.

Participants in both studies reported here described a rewarding experience as positive reinforcement in the form of congratulations for achievements (such as recording a no drinking day), recognition for actions (such as setting a goal), and the provision of feedback about progress toward their goals. The app was considered beneficial when it reassured participants about the trustworthiness of the information provided and spoke to them in a friendly, informal, and non-judgmental tone. Doubts about the benefits of the app, for example, how certain modules might help reduce consumption, were assuaged when participants understood more about why these modules were theorized to work.

### Differences between Studies

#### Feeling “Lost” and Unsure of What to Do Next

The third theme identified, that of “feeling lost and unsure what to do next,” was identified only among participants in the first study. Participants in the second study reported being able to navigate through the app without great difficulty, perhaps because repeated use resolved their initial confusion. However, users tend not to use new apps repeatedly; more than half of the apps downloaded are used less than five times (100). Therefore, it is not safe to assume that users will resolve issues of initial use without help. The commercial world addresses these problems with a process known as onboarding (101). Onboarding helps users become familiar with a technology and learn how its use might benefit them. It often takes the form of messages that guide users through the various elements on a screen or a stepped guide that walks users through the process of first using the app. Almost all participants in the first study requested a stepped guide be provided to help them first use the app, and many asked that guidance be provided about using elements throughout.

#### “Do Not Overwhelm Me” and “Blame Myself, Not the App, If It’s Too Hard to Use”

Two subthemes were identified only in the “think aloud” study: “Do not overwhelm me” and “Blame myself, not the app, if it’s too hard to use.” Participants in the “think aloud” study expressed concern that the range of options in the app might present an overwhelming amount of choice, a concern that corresponds with the theory that an excess of choice can inhibit action (102). Given that this subtheme was not identified among experienced users in the interview study, it is possible that people managed issues of overwhelm by using only the modules they found useful, a strategy some participants in the “think aloud” study had indeed proposed adopting. The presence of the “Blame myself, not the app, if it’s too hard to use” theme in only the “think aloud” study may also be explained by the ability of experienced users to solve problems with use. Alternatively, it is possible that the reason that both subthemes were not found in the interview study is because users who experienced these issues had stopped using the app and so did not respond to invitations to participate.

#### “Update Me on How I Am Doing” and “Be Friendly and Funny”

The subtheme “Update me on how I am doing” could only have been identified in the interview study with experienced users (and not the “think aloud” study of first-time users) because feedback about progress requires repeated use of the app. It is unclear why the subtheme “Be friendly and funny” was identified only in the “think aloud” study. However, one person in the interview study commented that the friendliness of the app increased with use, and evidence suggests that use of a system can increase user satisfaction with that system (103).

### Strengths and Limitations

A strength of the current evaluation was the use of two distinct approaches to usability. The first study identified issues with initial use, and the second identified issues with repeated use. Identifying and addressing both types of issue are essential if engagement with the DBCI is to be secured. In addition, the combination of findings from both studies allowed issues common to both first time and repeated use to be identified and given priority. This is important given the likelihood that limited timescales and budget will prevent all possible improvements arising from usability studies from being implemented.

A limitation of the study concerned the representativeness of the sample. A number of participants for the “think aloud” study were recruited from convenience sample of members of staff at a London university and their family and friends, the views of whom may not represent those of a typical drinker. Attempts to ensure representativeness were made by ensuring all participants were seeking to reduce their alcohol consumption and had scores on the AUDIT-C questionnaire that represented potentially harmful levels of drinking. Representativeness for the second study was further increased by recruiting participants from users who had downloaded the app unbidden. A second limitation concerned the analysis. Steps to ensure that findings accurately summarized the extracts included multiple readings of interviews and use of a second researcher to verify coding. However, researchers with greater experience in qualitative analyses and/or the evidence on alcohol reduction may have reached additional and/or different conclusions. A third limitation concerned the findings. Many of the findings may be considered usability basics, but it was clear that these issues remained of central importance to users, despite a concerted effort to address them in the version evaluated in this study. Care should also be taken when generalizing these findings: this was a study of a particular alcohol reduction app whose BCTs were implemented in a particular way findings may not apply to other behavior change apps. Analysis was also limited because participants were only audio recorded; greater understanding may have been gained by video recording participants’ interactions with the app and analyzing their comments and actions together (104). Finally, some of the participants were known to the first author and were aware of his role in the app’s development. It is possible that demand characteristics (105) may have affected these participants’ views toward the app.

## Conclusion

First-time and experienced users want an alcohol reduction app to be easy, rewarding, and beneficial to use. An easy-to-use app would reduce user burden, offer ongoing help, and be esthetically pleasing. A rewarding and beneficial app would demonstrate credibility, provide positive reinforcement, and give feedback about progress. First-time users need particular help to become familiar with the app; experienced users need compelling reasons to continue its use.

## Author Contributions

DC was responsible for preparing the manuscript, conducting the interviews, analyzing the data, and writing up the results. CG assisted in analyzing the data and writing up the results. JB, RW, and SM provided feedback and comments on the manuscript.

## Conflict of Interest Statement

JB has received an unrestricted research grant from Pfizer related to smoking cessation. RW has received research funding and undertaken consultancy for companies that manufacture smoking cessation medications. The other authors declare no conflict of interest.

## References

[1] World Health Organization. Global Status Report on Alcohol and Health, 2014. (2014). Available from: http://apps.who.int/iris/handle/10665/112736

[2] Public Health England. Alcohol Treatment in England 2012-13. (2013). Available from: http://www.nta.nhs.uk/uploads/alcohol2012-13.pdf

[3] Anderson P Baumberg B Alcohol in Europe A Public Health Perspective. Drugs: Education, Prevention, and Policy. London (2006). Available from: http://informahealthcare.com/doi/abs/10.1080/09687630600902477

[4] Rehm J Mathers C Popova S Thavorncharoensap M Teerawattananon Y Patra J. Global burden of disease and injury and economic cost attributable to alcohol use and alcohol-use disorders. Lancet (2009) 373(9682):2223–33. 10.1016/S0140-6736(09)60746-7 19560604

[5] Kaner EFS Beyer F Dickinson HO Pienaar ED Campbell F Schlesinger C Effectiveness of brief alcohol interventions in primary care populations. Cochrane Database Syst Rev (2007) 2:CD004148. 10.1002/14651858.CD004148.pub3 17443541

[6] Purshouse RC Brennan A Rafia R Latimer NR Archer RJ Angus CR Modelling the cost-effectiveness of alcohol screening and brief interventions in primary care in England. Alcohol Alcohol (2013) 48(2):180–8. 10.1093/alcalc/ags103 23015608

[7] Angus C Latimer N Preston L Li J Purshouse R. What are the implications for policy makers? A systematic review of the cost-effectiveness of screening and brief interventions for alcohol misuse in primary care. Front Psychiatry (2014) 5:114. 10.3389/fpsyt.2014.00114 25225487 PMC4150206

[8] Brown J West R Angus C Beard E Brennan A Drummond C Comparison of brief interventions in primary care on smoking and excessive alcohol consumption: a population survey in England. Br J Gen Pract (2016) 66(642):e1–9. 10.3399/bjgp16X683149 26719481 PMC4684029

[9] Kaner E Brief alcohol intervention: time for translational research. Addiction (2010) 105(6):960–1. 10.1111/j.1360-0443.2009.02848.x 20659054

[10] Heather N Dallolio E Hutchings D Kaner E White M Implementing routine screening and brief alcohol intervention in primary health care: a Delphi survey of expert opinion. J Subst Use (2004) 9(2):68–85. 10.1080/14659890410001665014

[11] Taylor CB Luce KH Computer- and Internet-based psychotherapy interventions. Curr Dir Psychol Sci (2003) 12(1):18–22. 10.1111/1467-8721.01214

[12] Marlatt G. Harm reduction: come as you are. Addict Behav (1996) 21(6):779–88. 10.1016/0306-4603(96)00042-1 8904943

[13] Norman GJ Zabinski MF Adams MA Rosenberg DE Yaroch AL Atienza AA A review of eHealth interventions for physical activity and dietary behavior change. Am J Prev Med (2007) 33(4):336–45. 10.1016/j.amepre.2007.05.007 17888860 PMC2180189

[14] Cugelman B Thelwall M Dawes P. Online interventions for social marketing health behavior change campaigns: a meta-analysis of psychological architectures and adherence factors. J Med Internet Res (2011) 13(1):e17. 10.2196/jmir.1367 21320854 PMC3221338

[15] Eysenbach G. The law of attrition. J Med Internet Res (2005) 7:e11. 10.2196/jmir.7.1.e11 15829473 PMC1550631

[16] Helander E Kaipainen K Korhonen I Wansink B. Factors related to sustained use of a free mobile app for dietary self-monitoring with photography and peer feedback: retrospective cohort study. J Med Internet Res (2014) 16(4):e109. 10.2196/jmir.3084 24735567 PMC4004142

[17] Hoch D App Retention Improves – Apps Used Only Once Declines to 20%. (2014). Available from: http://localytics.com; http://info.localytics.com/blog/app-retention-improves

[18] Nones P Marketing fail? Too many mobile apps are deleted within days of downloading. Nones Notes. (2014). Available from: http://nonesnotes.com/2014/01/06/marketing-fail-too-many-mobile-apps-are-deleted-within-days-of-downloading/

[19] Baker TB Gustafson DH Shah D. How can research keep up with eHealth? Ten strategies for increasing the timeliness and usefulness of Ehealth research. J Med Internet Res (2014) 16(2):e36. 10.2196/jmir.2925 24554442 PMC3961695

[20] van Gemert-Pijnen JEWC Nijland N van Limburg M Ossebaard HC Kelders SM Eysenbach G A holistic framework to improve the uptake and impact of eHealth technologies. J Med Internet Res (2011) 13(4):e111. 10.2196/jmir.1672 22155738 PMC3278097

[21] O’Brien HL The influence of hedonic and utilitarian motivations on user engagement: the case of online shopping experiences. Interact Comput (2010) 22(5):344–52. 10.1016/j.intcom.2010.04.001

[22] Jordan PW. Human factors for pleasure in product use. Appl Ergon (1998) 29(1):25–33. 10.1016/S0003-6870(97)00022-7 9769086

[23] Kim YH Kim DJ Wachter K A study of mobile user engagement (MoEN): engagement motivations, perceived value, satisfaction, and continued engagement intention. Decis Support Syst (2013) 56:361–70. 10.1016/j.dss.2013.07.002

[24] Hassenzahl M Platz A Burmester M Lehner K Hedonic and ergonomic quality aspects determine a software’s appeal. Proc SIGCHI Conf Hum Factors Comput Syst – CHI ’00 (Vol. 2), New York, NY: ACM Press (2000). p. 201–8. Available from: http://portal.acm.org/citation.cfm?doid=332040.332432

[25] Bargas-avila JA Hornbaek K Old wine in new bottles or novel challenges? A critical analysis of empirical studies of user experience. Proc SIGCHI Conf Hum Factors Comput Syst (2011). p. 2689–98. Available from: http://portal.acm.org/citation.cfm?doid=1978942.1979336

[26] Law EL-C The measurability and predictability of user experience. Proceedings of the 3rd ACM SIGCHI Symposium on Engineering Interactive Computing Systems – EICS ’11; New York: ACM (2011). p. 1–10. 10.1145/1996461.1996485

[27] van der Heijden H User Acceptance of Hedonic Information Systems. MIS Q (2004). p. 695–704.

[28] Ghani JA Deshpande SP Task characteristics and the experience of optimal flow in human–computer interaction. J Psychol (1994) 128(4):381–91. 10.1080/00223980.1994.9712742

[29] Webster J Ho H Audience engagement in multimedia presentations. ACM SIGMIS Database (1997) 28(2):63–77. 10.1145/264701.264706

[30] Hedegaard S Simonsen JG Extracting usability and user experience information from online user reviews. Proceedings of the SIGCHI Conference on Human Factors in Computing Systems – CHI ’13; New York: ACM (2013). p. 2089–98. 10.1145/2470654.2481286

[31] Yardley L Spring BJ Riper H Morrison LG Crane DH Curtis K Understanding and promoting effective engagement with digital behavior change interventions. Am J Prev Med (2016) 51(5):833–42. 10.1016/j.amepre.2016.06.015 27745683

[32] Yardley L Morrison L Bradbury K Muller I. The person-based approach to intervention development: application to digital health-related behavior change interventions. J Med Internet Res (2015) 17(1):e30. 10.2196/jmir.4055 25639757 PMC4327440

[33] Goh KN Chen YY Lai FW Daud SC Sivaji A Soo ST A comparison of usability testing methods for an E-commerce website: a case study on a Malaysia online gift shop. 2013 10th International Conference on Information Technology: New Generations IEEE (2013). p. 143–50. Available from: http://ieeexplore.ieee.org/articleDetails.jsp?arnumber=6614302

[34] Eveland WP Dunwoody S Examining information processing on the World Wide Web using think aloud protocols. Media Psychol (2000) 2(3):219–44. 10.1207/S1532785XMEP0203_2

[35] Ericsson KA Simon HA Verbal reports as data. Psychol Rev (1980) 87(3):215–51. 10.1037/0033-295X.87.3.215

[36] Nielsen J Estimating the number of subjects needed for a thinking aloud test. Int J Hum Comput Stud (1994) 41(3):385–97. 10.1006/ijhc.1994.1065

[37] Faulkner L. Beyond the five-user assumption: benefits of increased sample sizes in usability testing. Behav Res Methods Instrum Comput (2003) 35(3):379–83. 10.3758/BF03195514 14587545

[38] Lew P Olsina L Relating user experience with MobileApp quality evaluation and design. Lect Notes Comput Sci (2013) 8295:253–68. 10.1007/978-3-319-04244-2_23

[39] Zapata BC Fernández-Alemán JL Idri A Toval A. Empirical studies on usability of mHealth apps: a systematic literature review. J Med Syst (2015) 39(2):1. 10.1007/s10916-014-0182-2 25600193

[40] Nayebi F Desharnais J-M Abran A The state of the art of mobile application usability evaluation. 2012 25th IEEE Can Conf Electr Comput Eng (2012). p. 1–4. Available from: http://ieeexplore.ieee.org/lpdocs/epic03/wrapper.htm?arnumber=6334930

[41] Michie S Whittington C Hamoudi Z Zarnani F Tober G West R. Identification of behaviour change techniques to reduce excessive alcohol consumption. Addiction (2012) 107(8):1431–40. 10.1111/j.1360-0443.2012.03845.x 22340523

[42] Kaner EFS Beyer FR Brown J Crane D Garnett C Hickman M Personalised digital interventions for reducing hazardous and harmful alcohol consumption in community-dwelling populations. Cochrane Database Syst Rev (2015) (1):CD011479 10.1002/14651858.CD011479 PMC6483779 28944453

[43] Baharuddin R Singh D Razali R Usability dimensions for mobile applications – a review. Res J Appl Sci Eng Technol (2013) 5(6):2225–31.

[44] Nielsen Norman Group. Mobile Website and Application Usability, Nielsen Norman Group Report. Available from: http://www.nngroup.com/reports/mobile-website-and-application-usability/

[45] Lobo D Kaskaloglu K Kim CY Herbert S Web usability guidelines for smartphones: a synergic approach. Int J Infor Elec Eng (2011) 1(1):33–7. 10.7763/ijiee.2011.v1.5

[46] Coursaris CK Kim DJ A meta-analytical review of empirical mobile usability studies. J Usabilitiy Stud (2011) 6(3):117–71.

[47] Milward J Khadjesari Z Fincham-Campbell S Deluca P Watson R Drummond C. User preferences for content, features, and style for an app to reduce harmful drinking in young adults: analysis of user feedback in app stores and focus group interviews. JMIR mHealth uHealth (2016) 4(2):e47. 10.2196/mhealth.5242 27220371 PMC4897297

[48] Dulin PL Gonzalez VM Campbell K. Results of a pilot test of a self-administered smartphone-based treatment system for alcohol use disorders: usability and early outcomes. Subst Abus (2014) 35(2):168–75. 10.1080/08897077.2013.821437 24821354 PMC4019942

[49] Bernhardt JM Usdan S Mays D Arriola KJ Martin RJ Cremeens J Alcohol assessment using wireless handheld computers: a pilot study. Addict Behav (2007) 32(12):3065–70. 10.1016/j.addbeh.2007.04.012 17499442 PMC4388165

[50] Gajecki M Berman AH Sinadinovic K Rosendahl I Andersson C. Mobile phone brief intervention applications for risky alcohol use among university students: a randomized controlled study. Addict Sci Clin Pract (2014) 9(1):11. 10.1186/1940-0640-9-11 24985342 PMC4091647

[51] Hasin DS Aharonovich E Greenstein E. HealthCall for the smartphone: technology enhancement of brief intervention in HIV alcohol dependent patients. Addict Sci Clin Pract (2014) 9:5. 10.1186/1940-0640-9-5 24533631 PMC3943503

[52] Stoddard J Augustson E Smokers who use Internet and smokers who don’t: data from the Health Information and National Trends Survey (HINTS). Nicotine Tob Res (2006) 8(1):77–85. 10.1080/14622200601039147 17491174

[53] Parker RM Ratzan SC Lurie N. Health literacy: a policy challenge for advancing high-quality health care. Health Aff (2003) 22(4):147–53. 10.1377/hlthaff.22.4.147 12889762

[54] Boland VC Stockings EA Mattick RP McRobbie H Brown J Courtney RJ. The methodological quality and effectiveness of technology-based smoking cessation interventions for disadvantaged groups: a systematic review and meta-analysis. Nicotine Tob Res (2016) ntw391. 10.1093/ntr/ntw391 28034998

[55] Laing BY Mangione CM Tseng C-H Leng M Vaisberg E Mahida M Effectiveness of a smartphone application for weight loss compared with usual care in overweight primary care patients: a randomized, controlled trial. Ann Intern Med (2014) 161(10 Suppl):S5–12. 10.7326/M13-3005 25402403 PMC4422872

[56] Kauer SD Reid SC Crooke AHD Khor A Hearps SJC Jorm AF Self-monitoring using mobile phones in the early stages of adolescent depression: randomized controlled trial. J Med Internet Res (2012) 14(3):e67. 10.2196/jmir.1858 22732135 PMC3414872

[57] Grittner U Kuntsche S Graham K Bloomfield K. Social inequalities and gender differences in the experience of alcohol-related problems. Alcohol Alcohol (2012) 47(5):597–605. 10.1093/alcalc/ags040 22542707 PMC3417684

[58] Beard E Brown J West R Holmes J Kaner E Meier P Characterising the alcohol harm paradox: a population-based survey of adults in England. Lancet (2015) 386:S23 10.1016/S0140-6736(15)00861-2 PMC5040414 27682619

[59] Brown J Michie S Geraghty AWA Miller S Yardley L Gardner B A pilot study of StopAdvisor: a theory-based interactive internet-based smoking cessation intervention aimed across the social spectrum. Addict Behav (2012) 37(12):1365–70. 10.1016/j.addbeh.2012.05.016 22795643

[60] Bush K Kivlahan DR McDonell MB Fihn SD Bradley KA The AUDIT alcohol consumption questions (AUDIT-C): an effective brief screening test for problem drinking. Arch Intern Med (1998) 158(16):1789–95. 10.1001/archinte.158.16.1789 9738608

[61] Garnett C Development and Evaluation of a Theory- and Evidence-Based Smartphone App to Help Reduce Excessive Alcohol Consumption. University College London (2017).

[62] Crane D Development and Evaluation of a Smartphone App to Reduce Excessive Alcohol Consumption: Self-Regulatory Factors. University College London (2017).

[63] Braun V Clarke V Using thematic analysis in psychology. Qual Res Psychol (2006) 3(2):77–101. 10.1191/1478088706qp063oa

[64] Yardley L Miller S Teasdale E Little P Using mixed methods to design a web-based behavioural intervention to reduce transmission of colds and flu. J Health Psychol (2011) 16(2):353–64. 10.1177/1359105310377538 20929941

[65] Cafazzo JA Casselman M Hamming N Katzman DK Palmert MR. Design of an mHealth app for the self-management of adolescent type 1 diabetes: a pilot study. J Med Internet Res (2012) 14(3):e70. 10.2196/jmir.2058 22564332 PMC3799540

[66] Dennison L Morrison L Conway G Yardley L. Opportunities and challenges for smartphone applications in supporting health behavior change: qualitative study. J Med Internet Res (2013) 15(4):e86. 10.2196/jmir.2583 23598614 PMC3636318

[67] Morrison L Moss-Morris R Michie S Yardley L Optimizing engagement with Internet-based health behaviour change interventions: comparison of self-assessment with and without tailored feedback using a mixed methods approach. Br J Health Psychol (2013) 19(4):839–55. 10.1111/bjhp.12083 24308806 PMC4231218

[68] Davis FD Perceived ease of use, and user acceptance of information technology. MIS Q (1989) 13(3):319–40. 10.2307/249008

[69] Lyles CR Harris LT Le T Flowers J Tufano J Britt D Qualitative evaluation of a mobile phone and web-based collaborative care intervention for patients with type 2 diabetes. Diabetes Technol Ther (2011) 13(5):563–9. 10.1089/dia.2010.0200 21406018

[70] Rabin C Bock B Desired features of smartphone applications promoting physical activity. Telemed J E Health (2011):801–3. 10.1089/tmj.2011.0055 22010977

[71] Kim J Park H-A Development of a health information technology acceptance model using consumers’ health behavior intention. J Med Internet Res (2012) 14(5):e133 10.2196/jmir.2143 23026508 PMC3510715

[72] O’Brien HL Toms EG What is user engagement? A conceptual framework for defining user engagement with technology. J Am Soc Inf Sci Tec (2008) 59(6):938–55. 10.1002/asi.20801

[73] Thomas JG Wing RR. Health-e-call, a smartphone-assisted behavioral obesity treatment: pilot study. JMIR Mhealth and Uhealth (2013) 1(1):e3. 10.2196/mhealth.2164 25100672 PMC4114436

[74] Eysenbach G Kohler C. How do consumers search for and appraise health information on the World Wide Web? Qualitative study using focus groups, usability tests, and in-depth interviews. BMJ (2002) 324(7337):573–7. 10.1136/bmj.324.7337.573 11884321 PMC78994

[75] Krug S Don’t Make Me Think! A Common Sense Approach to Web Usability. India: Pearson Education (2005).

[76] Krebs P Duncan DT. Health app use among us mobile phone owners: a national survey. JMIR mHealth uHealth (2015) 3(4):e101. 10.2196/mhealth.4924 26537656 PMC4704953

[77] Atkinson NL Gold RS. The promise and challenge of ehealth interventions. Am J Health Behav (2002) 26(6):494–503. 10.5993/AJHB.26.6.10 12437024

[78] Gelso CJ Carter JA The relationship in counseling and psychotherapy: components, consequences, and theoretical antecedents. Couns Psychol (1985) 13(2):155–243. 10.1177/0011000085132001

[79] Lambert MJ Barley DE Research summary on the therapeutic relationship and psychotherapy outcome. Psychother Theory Res Pract Train (2001) 38(4):357–61. 10.1037/0033-3204.38.4.357

[80] Dishman RK Determinants of participation in physical activity. In: Bouchard C Shephard RJ Stevens T Sutton JR McPherson BD, editors. Exercise, Fitness and Health: A Consensus of Current Knowledge. Champaign, IL: Human Kinetics Publishers (1990). p. 78–101.

[81] Tsai AG. Systematic review: an evaluation of major commercial weight loss programs in the United States. Ann Intern Med (2005) 142(1):56. 10.7326/0003-4819-142-1-200501040-00012 15630109

[82] Hughes JR Keely J Naud S. Shape of the relapse curve and long-term abstinence among untreated smokers. Addiction (2004) 99(1):29–38. 10.1111/j.1360-0443.2004.00540.x 14678060

[83] Moos RH Moos BS. Rates and predictors of relapse after natural and treated remission from alcohol use disorders. Addiction (2006) 101(2):212–22. 10.1111/j.1360-0443.2006.01310.x 16445550 PMC1976118

[84] Shiffman S Hickcox M Paty JA Gnys M Kassel JD Richards TJ The abstinence violation effect following smoking lapses and temptations. Cognit Ther Res (1997) 21(5):497–523. 10.1023/A:1021853301255

[85] Muraven M Collins RL Morsheimer ET Shiffman S Paty JA. The morning after: limit violations and the self-regulation of alcohol consumption. Psychol Addict Behav (2005) 19(3):253–62. 10.1037/0893-164X.19.3.253 16187803

[86] Wrosch C Scheier MF Miller GE Schulz R Carver CS. Adaptive self-regulation of unattainable goals: goal disengagement, goal reengagement, and subjective well-being. Pers Soc Psychol Bull (2003) 29:1494–508. 10.1177/0146167203256921 15018681

[87] Carver CS Scheier MF Chapter 1: Self-regulation of action and affect. In: Vohs KD Baumeister RF, editors. Handbook of Self-Regulation, Second Edition: Research, Theory, and Applications. New York: Guilford Press (2010). p. 3–21.

[88] Skinner BF The behavior of organisms: an experimental analysis. Psychol Rec (1938):486 Available from: http://opensiuc.lib.siu.edu/tpr/vol47/iss4/5/

[89] Thorndike EL The law of effect. Am J Psychol (1927) 39(1/4):212–22. 10.2307/1415413

[90] Rothman AJ Toward a theory-based analysis of behavioral maintenance. Health Psychol (2000) 19:64–9. 10.1037/0278-6133.19.Suppl1.64 10709949

[91] Tang J Abraham C Stamp E Greaves C How can weight-loss app designers’ best engage and support users? A qualitative investigation. Br J Health Psychol (2015) 20(1):151–71. 10.1111/bjhp.12114 25130682

[92] McCurdie T Taneva S Casselman M Yeung M McDaniel C Ho W mHealth consumer apps: the case for user-centered design. Biomed Instrum Technol (2012) 46:49–56. 10.2345/0899-8205-46.s2.49 23039777

[93] Boswell W Why Users Uninstall Apps. Intel Developer Zone (2013). Available from: https://software.intel.com/en-us/blogs/2013/11/14/why-users-uninstall-apps

[94] VentureBeat. Five Reasons Users Uninstall Mobile Apps. (2013). Available from: http://venturebeat.com/2013/06/03/five-reasons-users-uninstall-mobile-apps/

[95] Lister C West JH Cannon B Sax T Brodegard D. Just a fad? Gamification in health and fitness apps. J Med Internet Res (2014) 16(8):e9. 10.2196/games.3413 25654660 PMC4307823

[96] Direito A Dale LP Shields E Dobson R Whittaker R Maddison R. Do physical activity and dietary smartphone applications incorporate evidence-based behaviour change techniques? BMC Public Health (2014) 14(1):646. 10.1186/1471-2458-14-646 24965805 PMC4080693

[97] Edwards EA Lumsden J Rivas C Steed L Edwards LA Thiyagarajan A Gamification for health promotion: systematic review of behaviour change techniques in smartphone apps. BMJ Open (2016) 6(10):e012447. 10.1136/bmjopen-2016-012447 27707829 PMC5073629

[98] Fogg B Persuasive technology: using computers to change what we think and do. Ubiquity (2002). 10.1145/764008.763957

[99] Crane D Garnett C Brown J West R Michie S. Behavior change techniques in popular alcohol reduction apps: content analysis. J Med Internet Res (2015) 17(5):e118. 10.2196/jmir.4060 25977135 PMC4468601

[100] O’Connell C 23% of Users Abandon an App After One Use. (2016). Available from: http://localytics.com; http://info.localytics.com/blog/23-of-users-abandon-an-app-after-one-use

[101] Germaine Satia. Mobile Onboarding: A Beginner’s Guide. Smashing Magazine (2014). Available from: https://www.smashingmagazine.com/2014/08/mobile-onboarding-beginners-guide/

[102] Schwartz B The Paradox of Choice: Why More Is Less. New York: Harper Perennial (2005).

[103] Ghahramani N Lendel I Haque R Sawruk K. User satisfaction with computerized order entry system and its effect on workplace level of stress. J Med Syst (2009) 33(3):199–205. 10.1007/s10916-008-9180-6 19408453

[104] Jaspers MW Steen T van den Bos C Geenen M. The think aloud method: a guide to user interface design. Int J Med Inform (2004) 73(11–12):781–95. 10.1016/j.ijmedinf.2004.08.003 15491929

[105] Orne MT Demand characteristics and the concept of quasi-controls 1. In: Rosenthal R Rosnow RL, editors. Artifacts in Behavioral Research (2009) p. 110–37. 10.1093/acprof:oso/9780195385540.003.0005

