# Supplementary material for: Factors Influencing Usability of a Smartphone App to Reduce Excessive Alcohol Consumption: Think Aloud and Interview Studies
Source: Front Public Health. 2017 Apr 3;5:39. doi: 10.3389/fpubh.2017.00039 (PMC5376568; doi:10.3389/fpubh.2017.00039)
Supplement: Supplementary file 1 [file data_sheet_1.docx]

## Appendix I – Tasks given and questions asked

## Appendix II Interview topics

### Study 1 – ‘Think aloud’

**Tasks**

1. Register for the app and complete baseline measures
2. Add drinks to the drinking diary
3. Set a goal
4. Create an action plan
5. Play the thanks/no thanks game
6. Try the identity module
7. Browse the app

**Questions**

1. What are your overall views toward the app?
2. Was there anything you particularly disliked?
3. Was there anything you found particularly hard to use?
4. Was there anything you particularly liked?
5. Was there anything you found particularly easy to use?
6. Anything you wanted to see there/expected to see there but didn’t?
7. Do you have any suggestions for how the app could be improved?
8. Are there any other comments you would like to make?

### Study 2 – Semi-structured interview

**Questions**

1. What made you choose this app in preference to others?
2. What was the registration process like? Was it too long? Or too intrusive?
3. What did you think of the feedback about your drinking? Did you believe it? What was your response to it?
4. What were your first impressions of the app?
5. What was your first impression of the dashboard?
6. What are your views of it now?
7. Do you remember what you did first when using the app and your views toward it?
8. Did you set any goals? How did you find the process?
9. Have you received any feedback yet? What do you think of it?
10. What do you think of the mood diary?
11. Did you set any action plans? How did you find the process? Have you found them useful?
12. How have you found the process of adding drinks?
13. Have you played the game? How did you find it? Were the instructions clear?
14. Have you tried the Identity section? How did you find it?
15. What do you think of the help section?
16. What are your overall views toward the app?
17. Was there anything you particularly disliked?
18. Was there anything you found particularly hard to use?
19. Was there anything you particularly liked?
20. Was there anything you found particularly easy to use?
21. Anything you wanted or expected to see but didn’t?
22. Do you have any other suggestions for how the app could be improved?
23. Are there any other comments you would like to make
